# Halogen bonding and chalcogen bonding mediated sensing

**DOI:** 10.1039/d2sc01800d

**Published:** 2022-05-11

**Authors:** Robert Hein, Paul D. Beer

**Affiliations:** Department of Chemistry, Chemistry Research Laboratory, University of Oxford Mansfield Road Oxford OX1 3TA UK paul.beer@chem.ox.ac.uk

## Abstract

Sigma–hole interactions, in particular halogen bonding (XB) and chalcogen bonding (ChB), have become indispensable tools in supramolecular chemistry, with wide-ranging applications in crystal engineering, catalysis and materials chemistry as well as anion recognition, transport and sensing. The latter has very rapidly developed in recent years and is becoming a mature research area in its own right. This can be attributed to the numerous advantages sigma–hole interactions imbue in sensor design, in particular high degrees of selectivity, sensitivity and the capability for sensing in aqueous media. Herein, we provide the first detailed overview of all developments in the field of XB and ChB mediated sensing, in particular the detection of anions but also neutral (gaseous) Lewis bases. This includes a wide range of optical colorimetric and luminescent sensors as well as an array of electrochemical sensors, most notably redox-active host systems. In addition, we discuss a range of other sensor designs, including capacitive sensors and chemiresistors, and provide a detailed overview and outlook for future fundamental developments in the field. Importantly the sensing concepts and methodologies described herein for the XB and ChB mediated sensing of anions, are generically applicable for the development of supramolecular receptors and sensors in general, including those for cations and neutral molecules employing a wide array of non-covalent interactions. As such we believe this review to be a useful guide to both the supramolecular and general chemistry community with interests in the fields of host–guest recognition and small molecule sensing. Moreover, we also highlight the need for a broader integration of supramolecular chemistry, analytical chemistry, synthetic chemistry and materials science in the development of the next generation of potent sensors.

## Abstract 2

Sigma–hole mediated detection of anions is rapidly emerging as a new paradigm in supramolecular sensor chemistry. Herein, we provide an overview of this field including halogen bonding and chalcogen bonding optical, electrochemical and other sensors.

## Graphical abstract

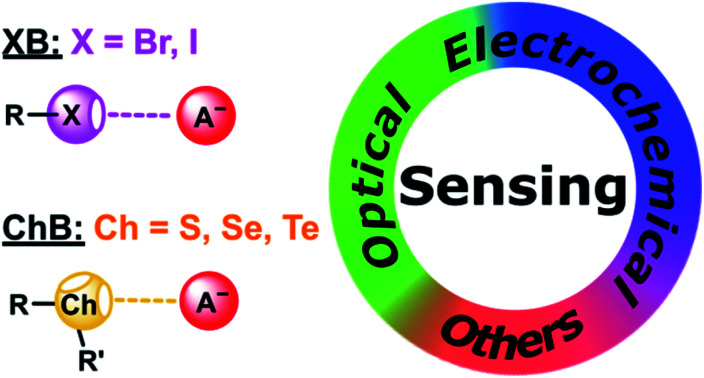

## 1. Introduction

Halogen bonding (XB) and chalcogen bonding (ChB), the non-covalent interactions between a positively charged region on a polarised, electron deficient halogen or chalcogen atom (the σ–hole) and a Lewis base, have emerged as powerful and potent additions to the supramolecular chemistry tool-box, being increasingly exploited in catalysis,^1–6^ crystal engineering^7–12^ and most notably molecular recognition.^13–21^ Stimulated by the importance of negatively charged species in a plethora of biological, industrial and environmental spheres, the XB-mediated recognition of anions, in particular, has significantly advanced in recent years.^14,16,22–24^ Due to their comparatively lower charge-density, stronger hydration as well as pH-dependence, the selective recognition of anions is significantly more challenging than cations, especially in aqueous environments.^25–27^ XB and ChB are ideally suited to address this challenge, as they typically imbue both enhanced selectivity and binding strength in comparison to hydrogen bonding (HB) analogues. This can be attributed to a variety of factors including a stricter adherence to a 180° binding geometry, lower solvent dependency, larger hydrophobicity and improved electronic tuneability.^14,16,28,29^

These combined advantages have facilitated sigma–hole mediated anion recognition in aqueous media, importantly including pure water.^30–32^ As a result, increasing attention is being directed at the application of this capability in transmembrane anion transport,^33–37^ ion extraction and remediation^38,39^ as well as anion sensing.^40–42^ The latter is highly relevant in many real-world scenarios, in particular environmental and healthcare monitoring. The reversible nature of non-covalent binding interactions in supramolecular host–guest systems is ideally suited for repeat and long-term sensing applications. To this end, the generation of anion sensors by integration of suitable optical or electrochemical reporter groups into hydrogen bond donor anion receptor structural frameworks has received enormous attention over the past few decades.^27,40–45^ In contrast, XB and ChB mediated anion sensing is a relatively new phenomenon, often demonstrating enhanced selectivity, binding strength and enhanced signal transducing capabilities in comparison to HB based sensing analogues.

Herein, we provide an overview of this field with a particular focus on the analytical performance of XB and ChB anion sensors. Where possible we contrast these sensors with analogous HB systems, highlighting the origins of enhanced XB and ChB sensing performances in the context of fundamental host–guest recognition principles.

## 2. Sigma–hole interactions and sensing

### 2.1 General overview and scope of the review

To date, ion-selective electrodes (ISEs) are the main employed anion sensors. Simple and cheap, they nevertheless often fail certain sensing criteria, as they possess thermodynamically limited sensitivities and require frequent (re)calibration.^46–49^ In order to satisfy a broad(er) range of application criteria, improved or complementary sensing approaches are thus highly desired. This has spurred on the development of a large range of alternative optical or electrochemical supramolecular anion sensors,^40–43,50^ wherein the use of σ–hole interactions is increasingly recognised as a particularly potent means to achieve higher degrees of selectivity and sensitivity as well as guest recognition and sensing in aqueous media.

This review gives a broad state-of-the art overview of all types of XB and ChB based sensors for anions, and also neutral Lewis bases and gases with a focus on their sensing performance in the context of sigma–hole recognition. Furthermore, we highlight how XB and ChB sensor systems contribute to fundamental aspects of supramolecular interactions as well as how they can aid in future developments in the fields of supramolecular host–guest chemistry and (ion) sensing in general.

**Fig. 1:**
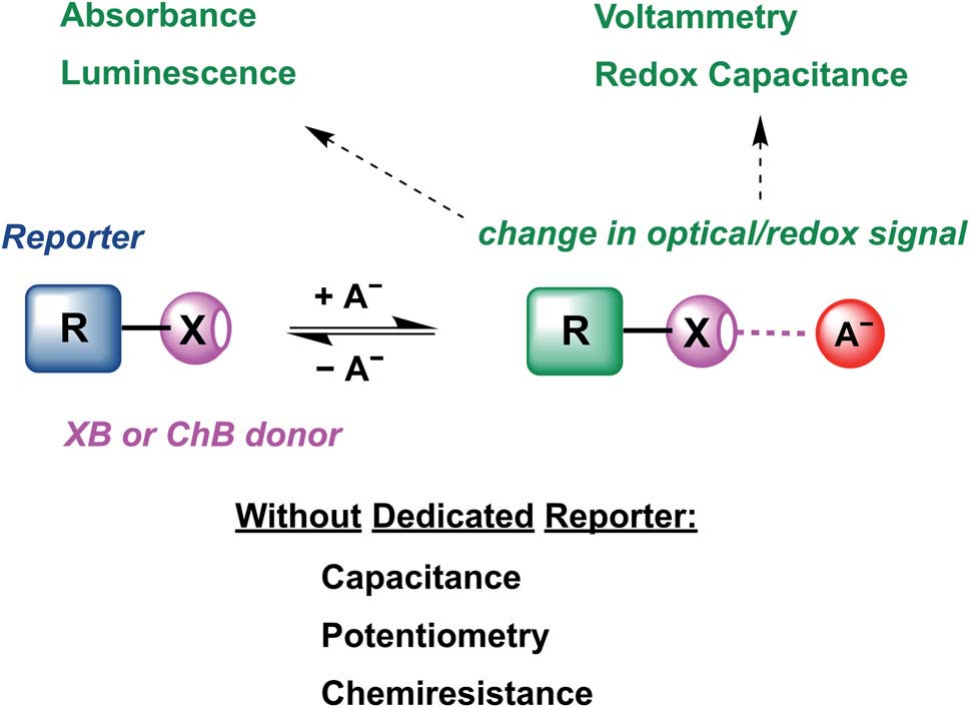
Schematic depiction of the most common supramolecular host–guest sensing approach based on the guest-binding induced modulation of the optical or electrochemical properties of a reporter group appended to a synthetic receptor. Shown here is the halogen bonding (XB) or chalcogen bonding (ChB)-mediated recognition of an anion as an archetypical Lewis base and its subsequent detection *via* various readouts.

The review begins with an introduction of the intrinsic properties of σ–hole interactions (Section 2.2) and their influence on relevant sensor parameters (Section 2.3). This is followed by a discussion of relevant examples of XB and ChB colorimetric and luminescent sensors (Section 3) as well as redox-active sensors (Section 4.1). The vast majority of these sensors are constructed by covalent appendage of reporter groups to XB or ChB receptors, whose optical or electrochemical properties are reversibly modulated in the presence of the bound guest analyte, as schematically shown in Fig. 1.

Nevertheless, a range of sensors omitting any dedicated reporter groups have been developed, including capacitive or chemiresistive sensors as discussed in Sections 4.2 (other electrochemical sensors) and 5 (other sensors). Finally, we provide an overview of future developments in the rapidly advancing field of sigma–hole mediated sensing and (an)ion sensing more generally (Section 6).

### 2.2 Brief introduction to sigma–hole interactions

**Fig. 2:**
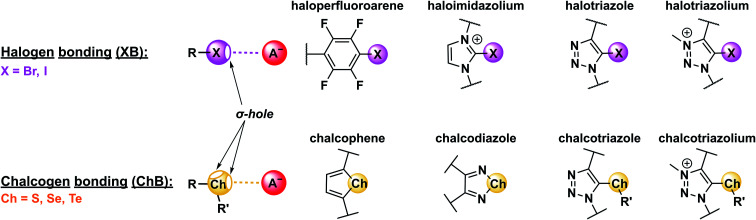
Schematic depiction of halogen (XB) and chalcogen bonding (ChB) interactions with an anion as archetypical Lewis base. Shown are also commonly employed XB and ChB donor motifs.

Appending a group 14–17 atom (X) with a substituent of higher electron-withdrawing capability (R) induces an anisotropic distribution of electron density such that a region of positive electrostatic potential is formed along the elongation of the R–X σ-bond (Fig. 2). This so-called σ–hole can then non-covalently interact with Lewis bases (LBs), forming halogen (XB), chalcogen (ChB), pnictogen (PnB) or tetrel bonds (TrB), for X = group 17, 16, 15 and 14, respectively.^14^ The strength of these σ–hole interactions is, of course, not only dependent on the nature and Lewis basicity of the acceptor, but is also highly tuneable *via* modification of the σ–hole donor scaffold (for examples see Fig. 2). Specifically, “deeper”, that is more electropositive, σ–holes are formed when the appended substituent is more electron-withdrawing. Similarly, larger, more polarisable and less electronegative donor atoms form more potent σ–holes, such that XB (and ChB) strength generally increases for the analogues descending the respective main group, *i.e.* I > Br > Cl and Te > Se > S.^14^††This trend is not strictly true for other σ–hole interactions such as PnB or TrB, for further information see ref. 14. Notably for XB the σ–hole is highly localised along the elongation of the R–X σ-bond, resulting in highly directional, linear non-covalent bond formation with Lewis bases, often exceeding 170° for the R–X⋯LB bond angle. These effects are primarily rationalised within an electrostatic bonding framework, that is based on the concept of the σ–hole. While conceptually useful and straight-forward, this description is not always sufficient to describe all sigma–hole properties. Specifically, a range of other factors, including polarisation, dispersion forces, hydrophobic effects and orbital interactions need to be considered in an accurate description of the nature of σ–hole bonding.^14^ This notably includes (partial) charge transfer from the Lewis base into the σ* orbital of the donor.^51^ This interaction between an occupied orbital of the Lewis base guest and an empty donor orbital is not only highly directional, but can also be a substantial driving force for sigma–hole bonding, giving rise to a significant degree of covalent character.^29,52–54^ This has also been experimentally observed for ChB interactions^29^ as well as halide-XB complexes.^55^ Recently, π-covalency was also shown to play a role in XB bond formation with halides.^56^ These observations may account for specific selectivity preferences, an improved sensor sensitivity as well as a generally lowered solvent dependence of σ–hole interactions in comparison to HB.^29,57,58^ For more in-depth discussions on the nature of σ–hole interactions the interested reader is referred to recent reviews.^14,51,59–61^

Of further note is that, depending on their hybridisation, ChB donors can form one (sp^2^) or two sigma holes (sp^3^). The “additional” substituent of sp^3^ hybridised ChB donors (in comparison to only one substituent on typical XB or HB donors), hereby presents a potent means of further tuning the σ–hole strength, or providing additional functionalities, such as optical or electrochemical reporter groups.^62^

### 2.3 Relevant sensor parameters

**Fig. 3:**
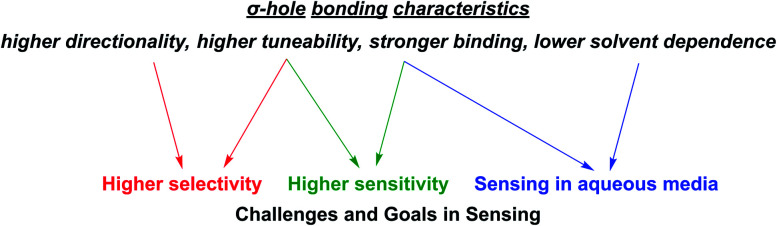
Important σ–hole bonding characteristics (in comparison to commonly employed HB and electrostatic interactions) and their impact on relevant challenges and goals in sensor development.

As alluded to above, an important goal in (supramolecular) sensor development is the capability of sensing in aqueous media. Their lower solvent dependence renders σ–hole interactions particularly potent in this regard. Similarly, the inherent characteristics of σ–hole interactions are highly relevant, and often beneficial, in addressing some of the other main goals in sensing and directly impacts most of the relevant sensing parameters as described in the following (Fig. 3).

#### 2.3.1 Selectivity

Achieving a high degree of selectivity is, at least from a supramolecular chemistry point of view, one of the most important goals in host–guest chemistry, and, by extension, the development of derived sensors. In this context, σ–hole interactions are, by virtue of their stricter geometric bonding preferences, ideal tools to address this challenge. As will be apparent from many examples in the following sections, XB and ChB mediated recognition and sensing is, in comparison to HB, often not only associated with contrasting selectivity patterns but also with an overall enhanced selectivity for a specific Lewis base (anion).

However, it also should be noted that highly selective recognition is not an absolute requirement for the construction of potent sensors. Firstly, depending on the specific application scenario, as well as the desired sensitivity, the co(existence) of certain levels of competing anions may be tolerated, in particular if their concentrations are low or their binding sufficiently weaker so as to not significantly compete with recognition and sensing of the target analyte.

In addition, the requirement for a high degree of selectivity of any *one* sensor may be overcome by employing an array of multiple different receptive sensor units such that a broader data set is obtained. From this, the desired sensory parameters, that is the concentration of one or multiple species, can then be obtained by, for example, deep-learning algorithms or more classical approaches such as linear discriminate analysis (LDA) or principal component analysis (PCA).^63–66^

This is an established approach to sense various analytes, including ions,^63–65,67–69^ but remains thus far unexplored in the context of sigma–hole mediated sensing. Sigma–hole interactions can offer much in this regard, as their typically contrasting selectivity and sensing patterns can complement that of more established sensors based on HB or electrostatic interactions.

#### 2.3.2 Sensitivity and limit of detection

**Fig. 4:**
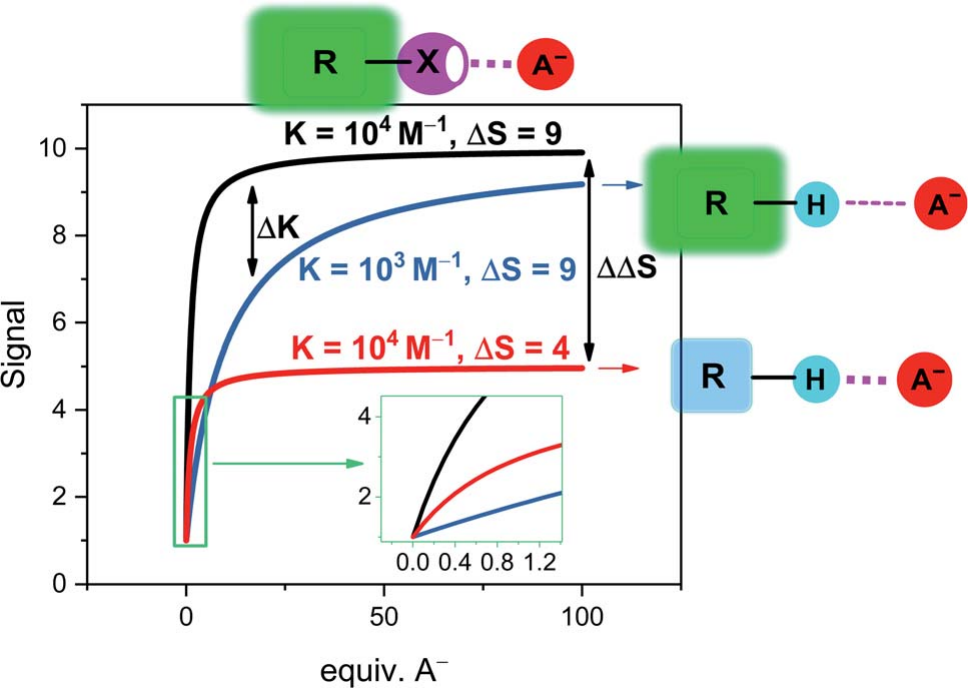
Simulated response isotherms of a generic non-covalent host–guest sensor based on the reporter group approach. The binding isotherms are simulated according to a simple 1 : 1 host–guest stoichiometric binding model with [H] = 100 μM and an initial free host signal of *S*_H_ = 1.^70^ Depicted are three exemplary cases: black line: strong binding (*K* = 10^4^ M^−1^) and a large signal change of the host–guest complex (*S*_HG_ = 10, Δ*S* = 9) as typically encountered in sigma–hole based sensors. The blue and red lines represent typical examples of less potent HB sensors in which either binding is weaker (blue line, *K* = 10^3^ M^−1^) or in which the signal magnitude is smaller (red line, *S*_HG_ = 5, Δ*S* = 4). This highlights the different mechanisms by which sigma–hole based sensors typically outperform related HB analogues in terms of sensitivity. Inset: magnification at low guest concentrations.

An important characteristic of any sensor is its analytical performance in terms of the linear/dynamic concentration range over which the analyte can be detected and its sensitivity, that is the slope of the calibration curve, *i.e.* how sensitive the sensor's signal is to a change in analyte concentration.‡‡The term “sensitivity” is ill-defined in the literature and can refer to both the LOD or the slope of the dose–response curve. Herein, we use the latter definition. A related, crucial parameter is the limit of detection (LOD), the lowest analyte concentration that can be reliably distinguished from the background noise. The LOD can be determined in different ways, but is commonly assessed as LOD = 3 × SD/*S* where SD represents the standard deviation of the response/blank and *S* the slope of the calibration curve (sensitivity). In many applications a low LOD is desired, which can be achieved by either lowering SD or increasing the sensitivity. The former is related to the methodology/instrumentation itself while the latter can be improved by chemical design of the sensor. Two considerations are of importance when aiming to improve probe sensitivity; stronger binding and a larger magnitude signal perturbation response from the reporter group upon binding, both of which are typically observed in sigma–hole based sensors (Fig. 4). Specifically, a larger binding constant will induce a higher degree of complexation, and associated signal change. Similarly, higher sensitivity will be achieved when the signal difference between the free host sensor and the host–guest complex is larger, *i.e.* when analyte binding induces larger electronic perturbations to the host, as is often the case in XB and ChB mediated sensors (see Fig. 4, larger Δsignal).

#### 2.3.3 Device integration

This often-overlooked requirement perhaps presents the most important, yet largely unaddressed challenge in translating ion sensors from the lab to real-life applications. In the development of real-life relevant sensors, a plethora of additional factors, including sample preparation, signal readout, stability, shelf-life, reusability, cost, simplicity, user-friendliness and many others have to be considered. While most of these are not directly relevant to the supramolecular design of ion sensors, some requirements, in particular device integration, are increasingly pertinent and should be considered in molecular design. Specifically, to date, the vast majority of reported ion sensors, both optical and electrochemical, operate in homogeneous solution, a setting in which the most important advantage of the host–guest sensing approach, its reversibility, cannot be easily exploited. This is because in homogeneous solution, the recovery and re-use of the supramolecular host probe is typically not feasible, such that the host–guest approach has no inherent merit over irreversible, reaction-based chemodosimeters. In order to leverage the reversibility of the non-covalent interaction with the analyte, the supramolecular host probe needs to be integrated into condensed matter such as interfaces, gels, polymers, metal–organic frameworks (MOFs) or (nano)particles. This enables facile continuous sensing by flowing of the sample solution over/through the aforementioned systems,^71,72^ but typically requires integration of anchor groups into the host scaffold. In this context sp^3^-hybridised ChB donor motifs are uniquely potent structural scaffolds, as the additional substituent allows for facile integration of (added) functionalities such as reporter or anchor motifs. However, this capability remains largely unexplored.^62,73^

## 3. Optical sensors

### 3.1 Colorimetric sensors

Perhaps the simplest example of a colour dependence in which XB plays a pivotal role is that of dissolved iodine; violet in the gas-phase as well as in non-polar solvents, while brownish in solvents of higher polarity. Over a century ago in 1903, Lachmann attributed this observation to formation of solvent·I_2_ adducts,^74^ whose wavelength of absorption is increasingly hypsochromically shifted for solvents with higher Lewis basicity.^15,75^

While this, and many of the following examples, can scarcely be considered useful real-life sensors, the use of UV-vis spectroscopy has, due to its simplicity and ubiquity, received enormous attention in the study of host–guest interactions, including sigma–hole bonding and sensing.^76^

In fact, many examples of XB-based anion receptors have been shown to undergo changes in absorbance upon anion recognition. This includes, for example, acyclic bimetallic rhenium(i)-containing XB iodotriazole receptors,^77^ acyclic and macrocyclic zinc(ii)-porphyrin iodotriazole XB hosts,^78,79^ XB iodopyridinium helicates^80^ and XB/HB iodoperfluoroaryl urea receptors.^81^

**Fig. 5:**
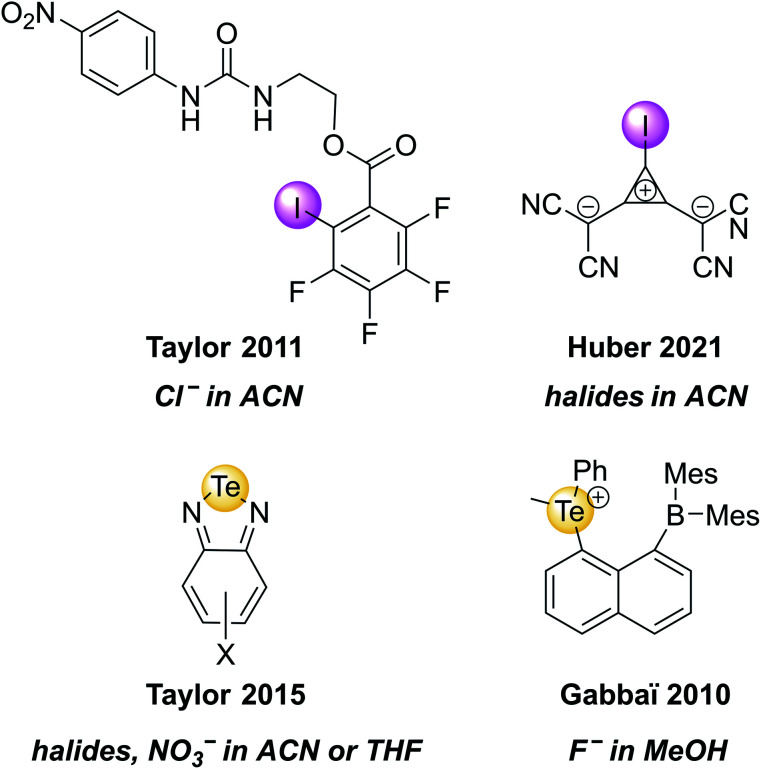
Selected examples of XB and ChB receptors that undergo changes in UV-vis absorbance upon exposure to the highlighted anions.

Recently, the groups of Huber and Rosokha specifically employed UV-vis spectroscopy to prove the formation of “anti-electrostatic” XB between halide anions and an anionic iodo-cyclopropenylium XB host in solution (Fig. 5).^82^ Similarly, UV-vis spectroscopy was employed to investigate guest binding in a range of ChB receptors.^36,62,83–86^ A few selected examples of such XB and ChB receptors that undergo changes in absorbance upon anion binding are shown in Fig. 5.

However, it must be noted that in the majority of these examples no systematic sensing studies were carried out and that the absorbance changes in most systems are only small; very few simple receptors undergo significant (naked-eye visible) changes.^78,87,88^

A powerful, but comparably rare strategy to induce large scale, naked-eye colorimetric changes upon anion recognition is the use of receptor co-conformational changes in mechanically interlocked molecules (MIMs) such as rotaxanes or catenanes upon guest binding.^41^ XB-mediated anion sensing *via* this strategy has been investigated by the Beer group in a range of [2] and [3]rotaxane shuttles.^89–91^

**Fig. 6:**
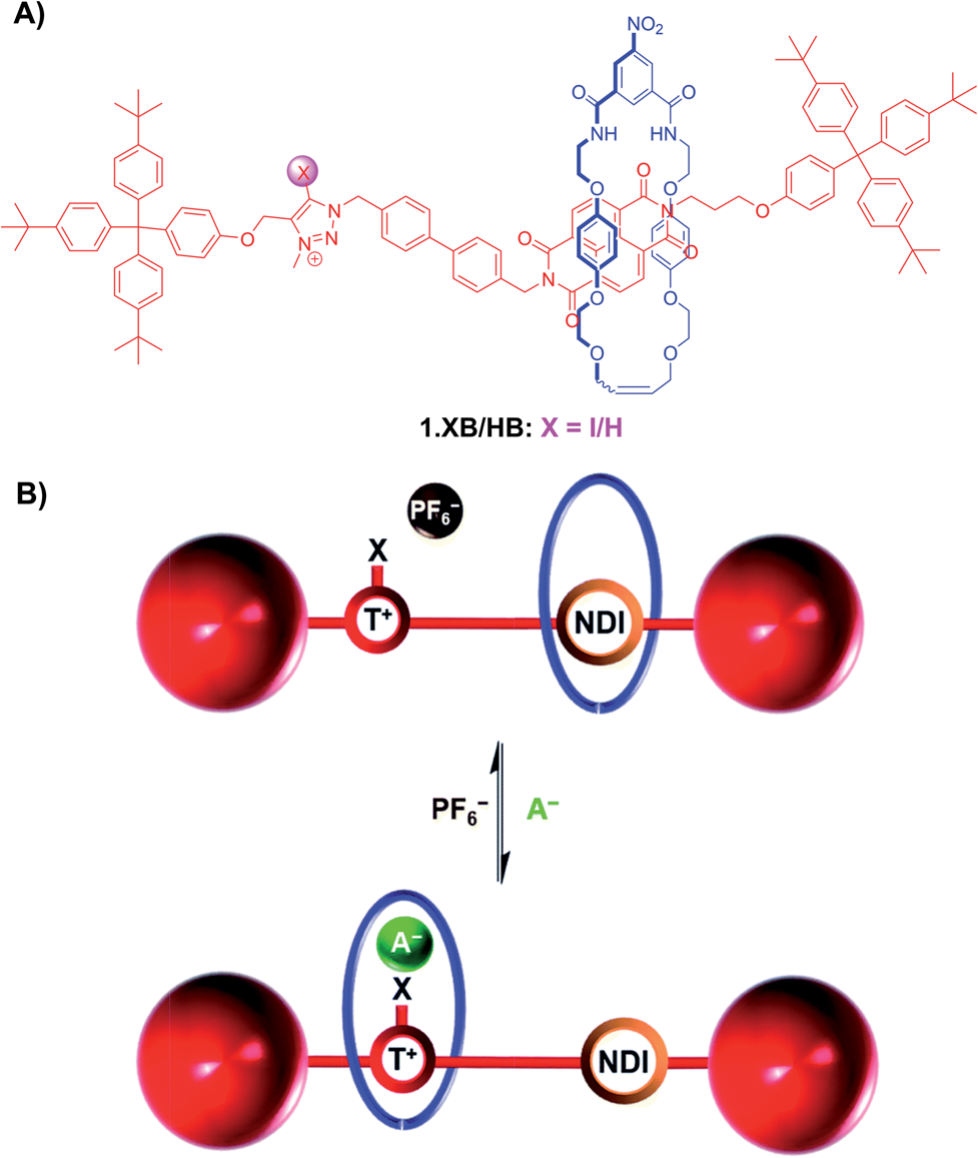
(A) Chemical structure of bistable (iodo)triazolium-NDI [2]rotaxanes 1.XB/HB. (B) Schematic depiction of anion recognition-induced shuttling of the macrocycle from the NDI to the triazolium, resulting in a naked-eye colour change from orange to colourless due to disruption of the donor–acceptor charge-transfer interactions between the hydroquinone-containing macrocycle and the NDI station. Reproduced from ref. 90 with permission from the Royal Society of Chemistry.

For instance, the bistable rotaxanes 1.XB/HB were developed as colorimetric anion sensors, undergoing halide binding-induced co-conformational changes and a concomitant colour change (Fig. 6).^90^ Specifically, in the absence of a coordinating anion guest, the electron-rich hydroquinone-containing macrocycle resides preferentially at the NDI station of the axle, resulting in an orange colour arising from donor–acceptor charge-transfer interactions. Upon addition of Cl^−^ or I^−^, convergent anion binding from the macrocycle's isophthalamide HB donors and the axle's (iodo)triazolium XB/HB donors induces a shuttling of the macrocycle to the triazolium station, thereby disrupting the donor–acceptor charge-transfer interactions resulting in a loss of colour.

Importantly, in the presence of 1 equiv. of halide anions in CDCl_3_ both 1.XB and 1.HB displayed an (almost) quantitative occupation of the triazolium station of at least 92% and 100%, respectively (Table 1). However, in the absence of coordinating anions, only the XB shuttle displayed a preferential occupation of the NDI station (62%), while in the HB system the macrocycle preferentially resided at the triazolium station (76%). This shows that the XB rotaxane is a superior shuttle, with larger changes in station occupancy upon anion recognition. This enhanced shuttling, and thus sensing, capability of 1.XB was also confirmed in more polar solvent systems. As shown in Table 1, in more competitive CDCl_3_/MeOD 4 : 1 and CDCl_3_/MeOD 1 : 1 both rotaxanes displayed reduced shuttling capabilities in the presence of halides, albeit with a generally superior performance of 1.XB. Notably in these competitive solvents the XB system showed a much more significant shuttling performance in the presence of I^−^ with an impressive 30% station occupancy change in CDCl_3_/MeOD 1 : 1, while 1.HB displayed a 10-fold worse shuttling behaviour (3% change, Table 1).

**Table 1:** Station occupancy of the macrocycle of bistable [2]rotaxanes 1.XB/HB of the triazolium (Trz) and NDI stations in various solvents and in the presence of 1 equiv. of different anions as determined by ^1^H NMR^90^

	Anion	1.XB			1.HB		
		Trz	NDI	Δ(A^−^–PF_6_^−^)	Trz	NDI	Δ(A^−^–PF_6_^−^)
CDCl_3_	PF_6_^−^	38%	62%	—	76%	24%	—
	Cl^−^	92%	8%	54%	100%	0%	24%
	I^−^	95%	5%	57%	100%	0%	24%
CDCl_3_/MeOD 4 : 1	PF_6_^−^	52%	48%	—	67%	33%	—
	Cl^−^	100%	0%	48%	87%	13%	20%
	I^−^	92%	8%	40%	70%	30%	3%
CDCl_3_/MeOD 1 : 1	PF_6_^−^	33%	67%	—	33%	67%	—
	Cl^−^	48%	52%	15%	49%	51%	16%
	I^−^	63%	37%	30%	36%	64%	3%

Building on these results, a more elaborate, structurally related XB/HB four station [3]rotaxane containing two triazolium and two NDI stations, as well as two hydroquinone-isophthalamide macrocycles was developed.^89^ Upon addition of Cl^−^ or NO_3_^−^ in CDCl_3_, the macrocycles undergo a concerted pincer-type shuttling motion from the peripheral NDI stations to the central triazolium stations and, as in the previous example, induce a colour change from orange to colourless. Binding of the smaller halide anion proceeds *via* 1 : 2 host–guest stoichiometric binding, while the larger, trigonal planar NO_3_^−^ binds strongly with a 1 : 1 stoichiometry, bridging the axle and both macrocycles. Of further note is not only the expectedly enhanced XB recognition performance of the XB [3]rotaxane, but also a rare NO_3_^−^ selectivity over Cl^−^ and a range of other oxoanions.

**Fig. 7:**
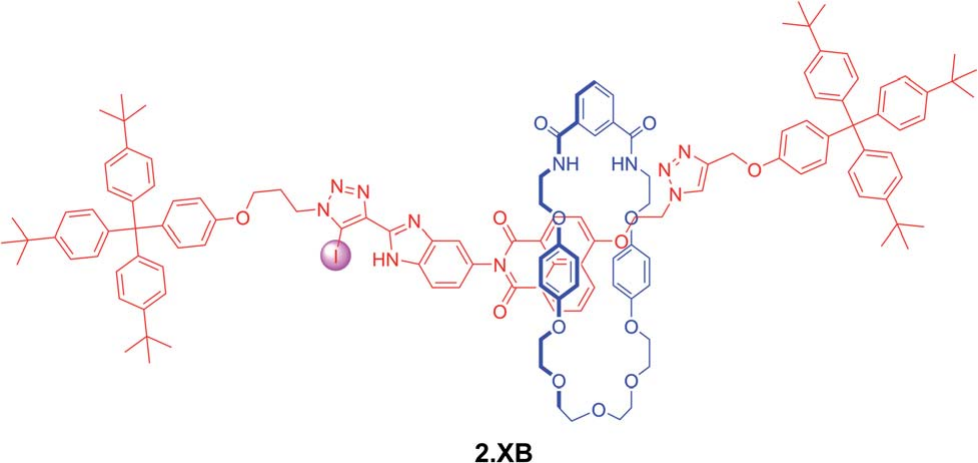
XB [2]rotaxane molecular shuttle 2.XB for colorimetric anion sensing in CHCl_3_. Only in the presence of a coordinating Cl^−^ anion as well as acid-induced protonation of the benzimidazole does the macrocycle shuttle towards the XB anion binding site, resulting in a disruption of the hydroquinone-NDI charge-transfer interaction and a concomitant loss of colour.

In a more recent example, Klein *et al.* prepared the bistable [2]rotaxane shuttle 2.XB for anion and pH dependent molecular motion and sensing (Fig. 7).^91^ In analogy to the previous examples, the hydroquinone-isophthalamide-containing macrocycle of the neutral, unprotonated rotaxanes preferentially resides on the axle electron deficient naphthalimide motif, resulting in a yellow colouration. In CDCl_3_ neither protonation of the benzimidazole nor presence of Cl^−^ alone induced any shuttling of the macrocycle. Only in the presence of both acid *and* anion was macrocycle translocation to the benzimidazolium–iodotriazole anion binding station observed, resulting in loss of colour as well as fluorescence emission increase. The rotaxane shuttle thus behaves as a molecular logic AND gate, requiring both a coordinating anion as well as anion binding enhancement *via* benzimidazole-protonation to function.

### 3.2 Luminescent sensors

In an effort to provide a more sensitive and useful sensor readout in comparison to the colorimetric sensors discussed above, the development of luminescent sigma–hole-based probes has gained significant attention in the last decade. To this end, a diverse range of XB, and to a much lesser extent ChB, acyclic, macrocyclic and interlocked receptor architectures have been endowed with various organic and transition-metal based luminescent motifs, providing a simple and highly sensitive means of sensing of various anions.^41–43^

#### 3.2.1 Halogen bonding luminescent sensors

**Fig. 8:**
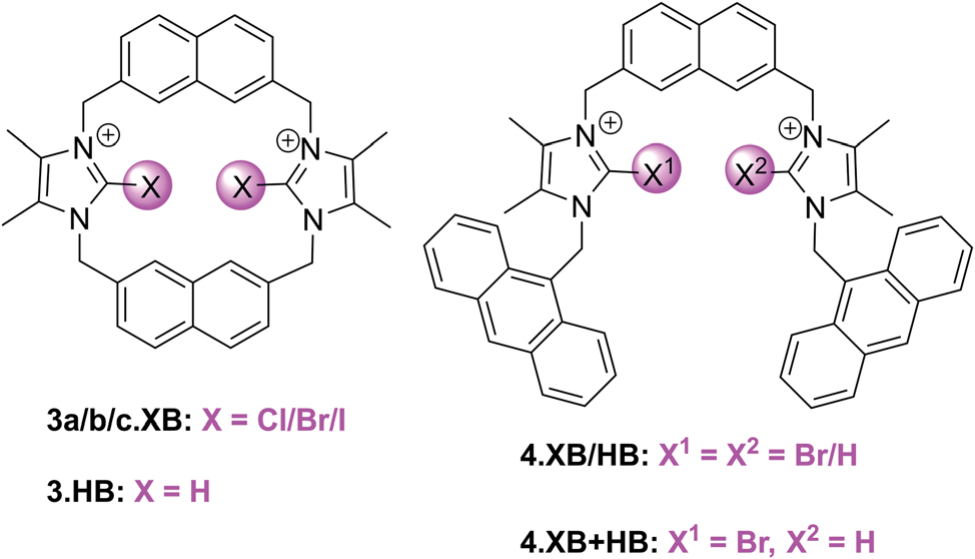
(Halo)imidazolium hosts for fluorescent anion sensing.

One of the first examples of a potent XB fluorescent anion sensor system was developed by Zapata *et al.* in 2012.^92^ The macrocyclic halo-imidazolium hosts 3a–c.XB (Fig. 8) were investigated as anion receptors and sensors in the highly competitive CH_3_OH/H_2_O 9 : 1 solvent system, wherein ^1^H NMR studies revealed strong 1 : 1 host–guest stoichiometric binding of Br^−^ and I^−^ to the *syn-*isomers of the bromo- and iodo-imidazolium hosts 3b.XB and 3c.XB (*K* > 10 000 M^−1^ for 3b.XB).

In contrast, a large range of oxoanions (H_2_PO_4_^−^, NO_3_^−^, SO_4_^2−^, AcO^−^, BzO^−^) as well as F^−^ and Cl^−^ did not bind to these hosts. Similarly, neither the weaker XB Cl-donor derivative 3a.XB nor the *anti* conformer of 3c.XB (capable of only forming one XB-anion interaction) bound any of the tested guests, while binding of the three halides Cl^−^, Br^−^ and I^−^ to the HB host 3.HB was very weak (<85 M^−1^).

**Fig. 9:**
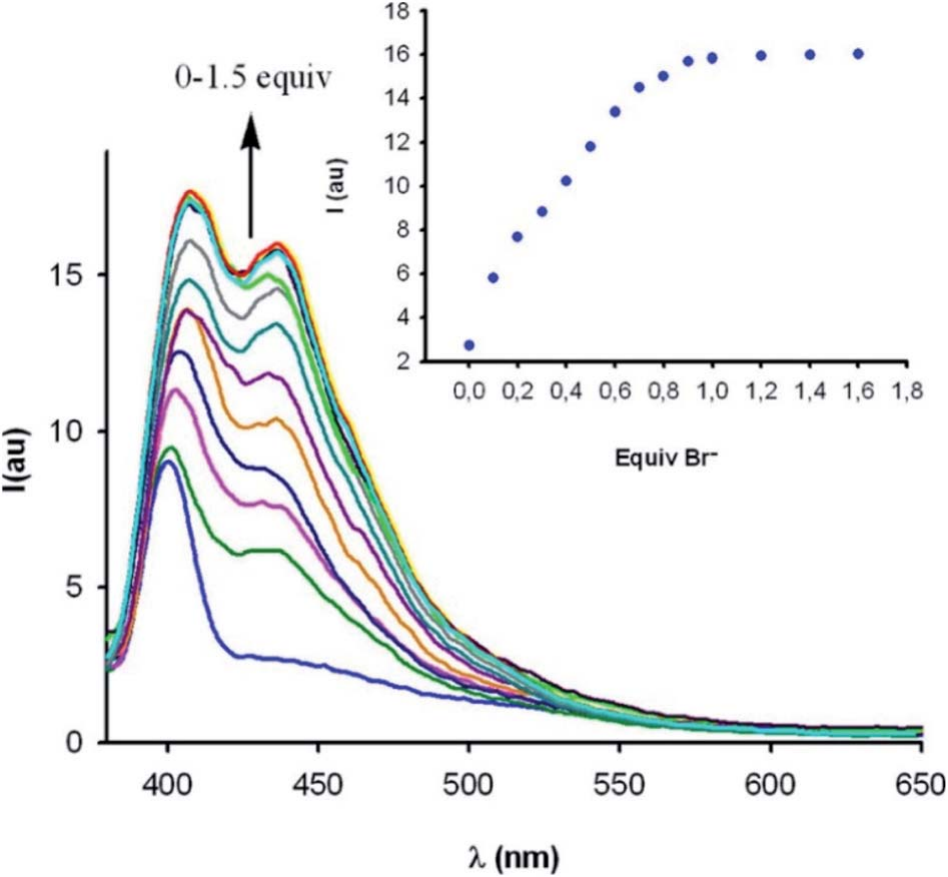
Fluorescence emission response of macrocyclic iodo-imidazolium host 3c.XB (10 μM) in CH_3_OH/H_2_O 9 : 1 in the presence of increasing concentrations of bromide. Reproduced with permission from ref. 92 copyright 2012 American Chemical Society.

Fluorescence sensing studies mirrored these trends; neither 3.HB nor 3a.XB responded to any anions, while significant enhancement of the naphthalene emission, in particular of the initially weaker low-energy band, was observed for both the bromo- and *syn* iodo-imidazolium hosts in the presence of Br^−^ and I^−^ (Fig. 9). Interestingly, 3b.XB displayed more significant emission enhancements of up to 5.8× in the presence of I^−^ (*K* = 63 100 M^−1^) than Br^−^ (2×, *K* = 2880 M^−1^), while the iodo-imidazolium receptor displayed preferential Br^−^ binding and enhancements (6.4×, *K* = 95 500 M^−1^), with weaker I^−^ binding (1.6×, *K* = 3710 M^−1^). This highlights the enormous potency of XB for tuneable, highly selective halide sensing in aqueous media.

Building on this motif, the groups of Caballero and Molina developed related, anthracene appended acyclic (bromo)imidazolium receptors 4.XB/HB as well as the mixed XB and HB receptor 4.XB+HB.^93,94^ In ACN 4.XB displayed no fluorescence changes in the presence of Cl^−^, Br^−^, I^−^ and various oxoanions, most notably HSO_4_^−^, AcO^−^ and BzO^−^, whereas F^−^ and SO_4_^2−^ induced significant fluorescence quenching.^93^ In contrast, HP_2_O_7_^3−^ induced notable emission turn-on, while in the presence of H_2_PO_4_^−^ a higher-wavelength emission band appears, arising from formation of anthracene excimers. As a result of this unique response pattern, this XB probe can thus selectively sense the dihydrogen phosphate anion, as further confirmed by competition experiments; only in the presence of 2 equiv. of HP_2_O_7_^3−^ or SO_4_^2−^ were changes in the H_2_PO_4_^−^-induced excimer band observed. Interestingly, 4.HB displayed only quenching in the presence of F^−^, SO_4_^2−^, HP_2_O_7_^3−^ and H_2_PO_4_^−^, with no appearance of excimer emission. The mixed 4.XB+HB receptor displays response patterns that are generally identical to those of the XB probe, with an additional moderate quenching response towards AcO^−^.^94^ In a later study a related tripodal bromoimidazolium anthracene receptor displayed similar selective detection of H_2_PO_4_^−^*via* the same excimer response mechanism.^95^ The same group also developed a tetra bromoimidazole-tetraphenylethylene as an ion-pair receptor, capable of selective emission turn-on sensing of HSO_4_^−^ in the presence of co-bound Zn^2+^ in ACN.^96^

In analogy to many of the other sensors described in the other sections of this review, the ubiquitous 5-iodo-1,2,3-triazole motif has been exploited in a large range of fluorescent XB anion sensors. For example, Zapata *et al.* developed a range of bis(halotriazolium-pyrene) hosts for the sensing of pyrophosphate and dihydrogenphosphate in acetone *via* pyrene excimer formation (akin to the above mentioned 4.XB system).^97^ Fluorescent H_2_PO_4_^−^ sensing in ACN was also reported by emission enhancements of a BINOL-bis(triazolium) system.^98^

**Fig. 10:**
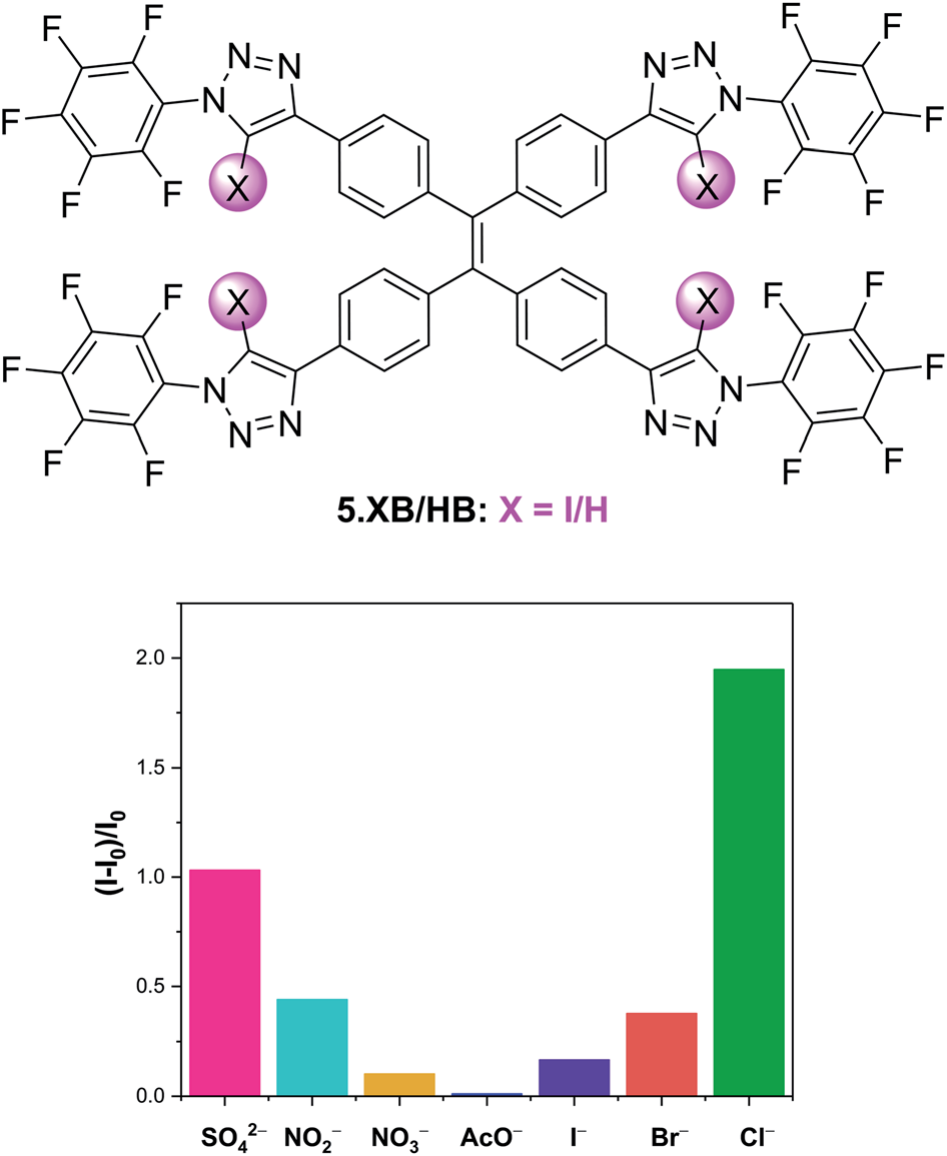
Relative emission intensity increase of 10 μM tetraphenylethene AIE anion sensor 5.XB in the presence of 10 equiv. of various anions in THF. Reproduced from ref. 100 under the terms of the CC BY license.

Aggregation-induced emission (AIE) has over the last two decades rapidly emerged as a new paradigm in a plethora of luminescence applications, but remains notably underexplored in the context of host–guest ion sensing.^99^ Recently, Docker and Shang *et al.* conducted a systematic binding and sensing study of a range of iodo-triazole appended tetraphenylethene (TPE) receptors as AIE platforms.^100^ The tetra-XB receptor 5.XB displayed expectedly strong Cl^−^ recognition in d_8_-THF and responded to various anions *via* significant fluorescent enhancements, with a notable halide selectivity, as shown in Fig. 10. The origins of this response were ascribed to anion binding-induced AIE, as supported by DLS and TEM measurements, confirming the presence of ≈100 nm sized particles only in the presence of anions. This fluorescent AIE response was notably not observed for the HB analogue 5.HB nor for a weaker XB donor receptor analogue containing phenyl instead of perfluorophenyl substituents. Similarly, the mono-XB TPE derivative displayed no significant emission response. In spite of strong halide binding by all three possible doubly-substituted XB TPE isomers, only the 1,1-diXB-ethene isomer exhibited chloride-induced AIE, highlighting the importance of the spatial orientation of XB donor sites to effect AIE.

The authors further demonstrated that the E and Z-derivatives of the other doubly-substituted 1,2-diXB-ethene XB TPE receptor can be interconverted by light, whereby the relative composition in the photostationary state is dependent on anion presence.

Another recent research focus in the development of XB fluorescent sensors has been their operation in (pure) aqueous environments. To this end Beer and co-workers have developed a range of water-soluble optically responsive XB receptors, including a benzene bis(iodotriazolium) host for emission turn-on sensing of ReO_4_^−^ in HEPES buffer (pH = 7.4)^32^ as well as a naphthalimide-appended XB foldamer receptor for sensing of I^−^ in water *via* fluorescence enhancement.^31^

**Fig. 11:**
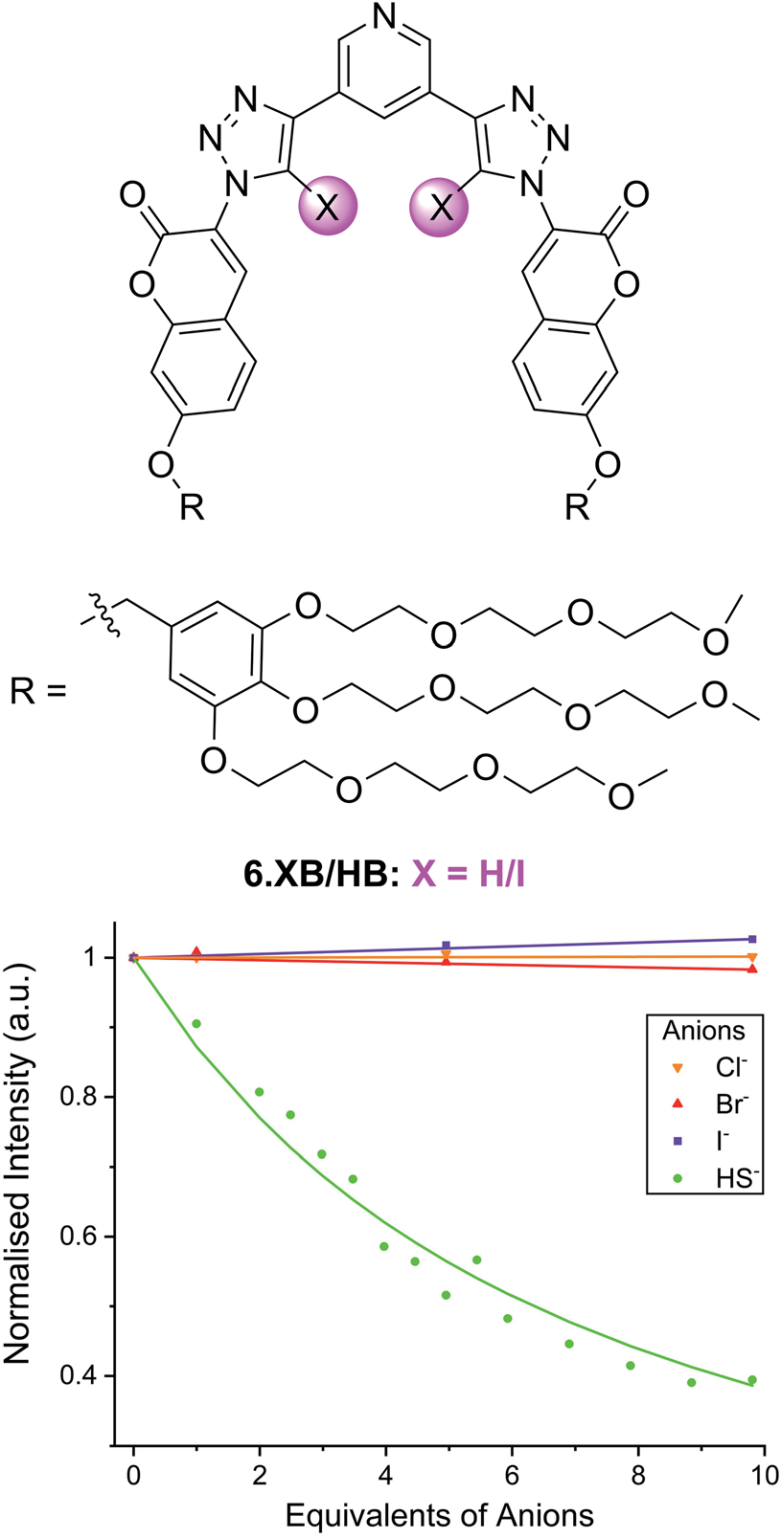
Fluorescence emission intensity response of 10 μM hydrosulfide-selective coumarin-containing 6.XB in the presence of 10 equiv. of various anions in 10 mM HEPES buffer (pH = 7.4). Reproduced from ref. 101 under the terms of the CC BY license.

In another example, the Beer group recently developed the XB coumarin-appended receptor 6.XB as a hydrosulfide (HS^−^) selective fluorescent probe.^101^ Formed upon dissolution of the toxic H_2_S gas, the sensing of HS^−^ remains a formidable challenge, in spite of its increasing relevance in environmental and medicinal settings.^102,103^ Thus far, the vast majority of HS^−^ sensors are irreversible optical chemodosimeters,^104,105^ while host–guest recognition of this anion remains largely underexplored.^106–108^ Receptor 6.XB not only presents a rare example of a reversible supramolecular HS^−^ host but is capable of selective sensing of this analyte in water.^101^ As shown in Fig. 11, addition of up to 10 equiv. of HS^−^ to a buffered solution of 6.XB induced notable coumarin emission quenching of up to 60%, while neither Cl^−^, Br^−^ nor I^−^ induced an appreciable response. While the XB sensor displayed strong HS^−^ binding (*K* = 16 500 M^−1^) and a sensitive fluorescence response (LOD = 14 μM), the HB analogue 6.HB did not display any anion detection capability. These findings were further corroborated by DFT and molecular dynamics simulations, highlighting a unique potency of XB for the recognition of HS^−^.

**Fig. 12:**
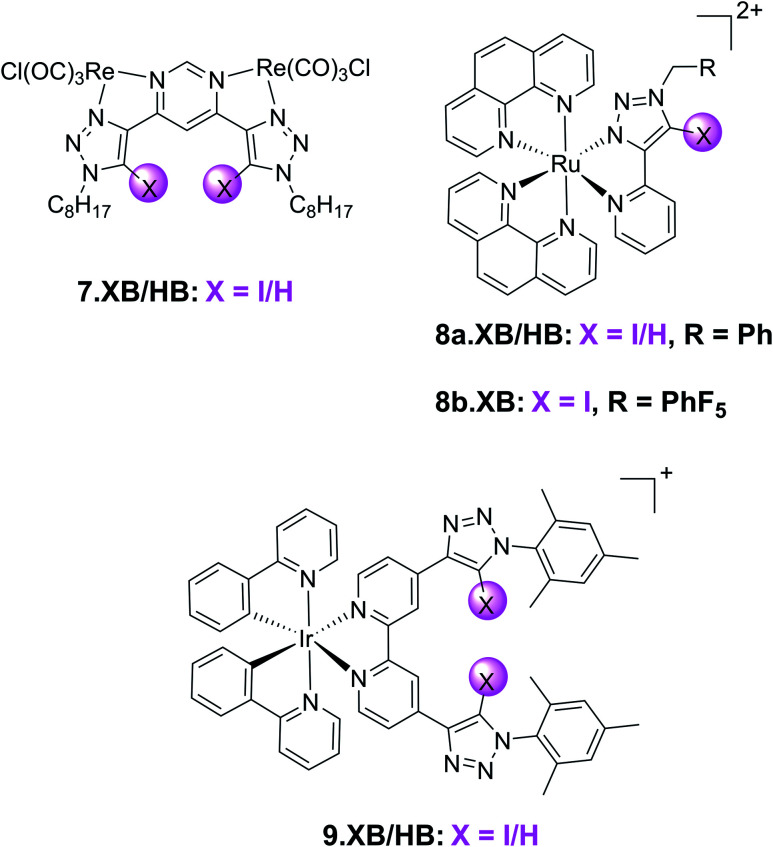
Chemical structures of acyclic transition metal-containing luminescent XB anion receptors.

In addition to the organic fluorophores discussed above, various organometallic and transition metal-based emissive XB sensors have been developed. Their typically modular synthesis and bright, highly tuneable emission profiles renders them potent motifs in optical ion sensors.^109^ This has been exploited in a range of XB fluorescent anion sensors, an early example of which is the neutral, bimetallic pyrimidine-(iodo)triazole 7.XB/HB system containing Re(i) carbonyl reporter groups (Fig. 12).^110^ Preorganisation and polarization of the triazole binding site by this organometallic motif enabled strong binding of a range of halides and oxoanions in 1 : 1 CDCl_3_/MeOD, with strongest binding observed for iodide at 7.XB with *K* > 10 000 M^−1^. Preliminary sensing studies in ACN revealed absorbance changes and luminescence enhancements of both receptors in the presence of the halides, phosphate and sulfate.

A similar design concept was exploited in receptors 8a–b.XB/HB by the group of Ghosh.^111,112^ In ACN 8a.XB displayed significant emission enhancement of the Ru(phen)_2_py-triazole MLCT band of 5 and 17-fold in the presence of HP_2_O_7_^3−^ and H_2_PO_4_^−^, respectively, while a large range of other oxoanions and halides did not induce significant responses.^111^ This correlated with stronger binding of the latter anion, with a 1 : 1 host–guest stoichiometric binding constant of *K* = 194 000 M^−1^, bound 3.5-fold more strongly than pyrophosphate. This was also confirmed by competition experiments; even in the presence of 10 equiv. of various competing anions the sensor response towards 1 equiv. of H_2_PO_4_^−^ was largely unaltered. In addition, the authors reported a notable increase in the MLCT luminescence lifetimes of the probe, from ≈6 ns of the free receptor to ≈34 and 109 ns in the presence of HP_2_O_7_^3−^ and H_2_PO_4_^−^, respectively. For the HB analogue 8a.HB the sensing performance towards these anions was expectedly reduced, with lower binding constants, larger LODs as well as reduced life-time enhancements. This is also reflected in a significant sensing performance for 8a.XB in up to 20% water in ACN (albeit with lower response magnitudes), while 8a.HB was incapable of sensing the phosphate anions in mixtures containing 10% or more water.

In a subsequent study, the same group also investigated the more polarized pentafluorophenyl appended receptor analogue 8b.XB.^112^ Unsurprisingly, the XB receptor displayed enhanced binding of both HP_2_O_7_^3−^ and H_2_PO_4_^−^ (*K* = 8.9 × 10^5^ and 2.76 × 10^6^ M^−1^ in ACN, respectively), over 10-fold larger than the benzyl-appended 8a.XB. In addition, 8b.XB also displayed a larger switch-on response of 25-fold in the presence of H_2_PO_4_^−^, while the enhancements in the presence of HP_2_O_7_^3−^ were comparably somewhat attenuated (3.6-fold increase). The LOD was low towards both anions (≈11 and 91 nM, respectively).

The first example of a XB receptor containing the ubiquitous, luminescent cyclometallated Ir(ppy)_2_-motif^113,114^ was reported by Schubert and co-workers in 2020.^115^ Containing an additional 4,4-bis-iodotriazole bipy ligand, 9.XB displayed significantly enhanced Cl^−^ binding (60 000 M^−1^) in comparison to its HB congener in ACN (5000 M^−1^). Both Br^−^ and OAc^−^ were also bound, albeit weaker. 9.XB exhibited a significant luminescence response towards chloride with a low LOD of 11 nM, while the perturbations induced by the other anions were notably smaller.

Acyclic fluorescent XB sensors based on other XB donor motifs include, for example, iodo-naphthoquinone receptors for sensing of SO_4_^2−^ in ACN^116^ and iodo-pyridinium receptors for sensing of various halides and oxoanions in DCM.^117^

#### 3.2.2 Interlocked luminescent XB sensors

As a result of their well-defined three-dimensional binding cavities, mechanically interlocked receptors have garnered significant attention in ion recognition and sensing.^40,41,50,118,119^ Both rotaxane and catenane hosts advantageously display enhanced binding strength and selectivities in comparison to acyclic or macrocyclic systems; mechanical bond effect properties that synergise particularly well with sigma–hole donors, as increasingly exploited in anion supramolecular chemistry.^30,120,121^ Unsurprisingly, significant attention has been directed at the incorporation of various reporter groups into these interlocked ion receptors, in particular luminescent reporters.

**Fig. 13:**
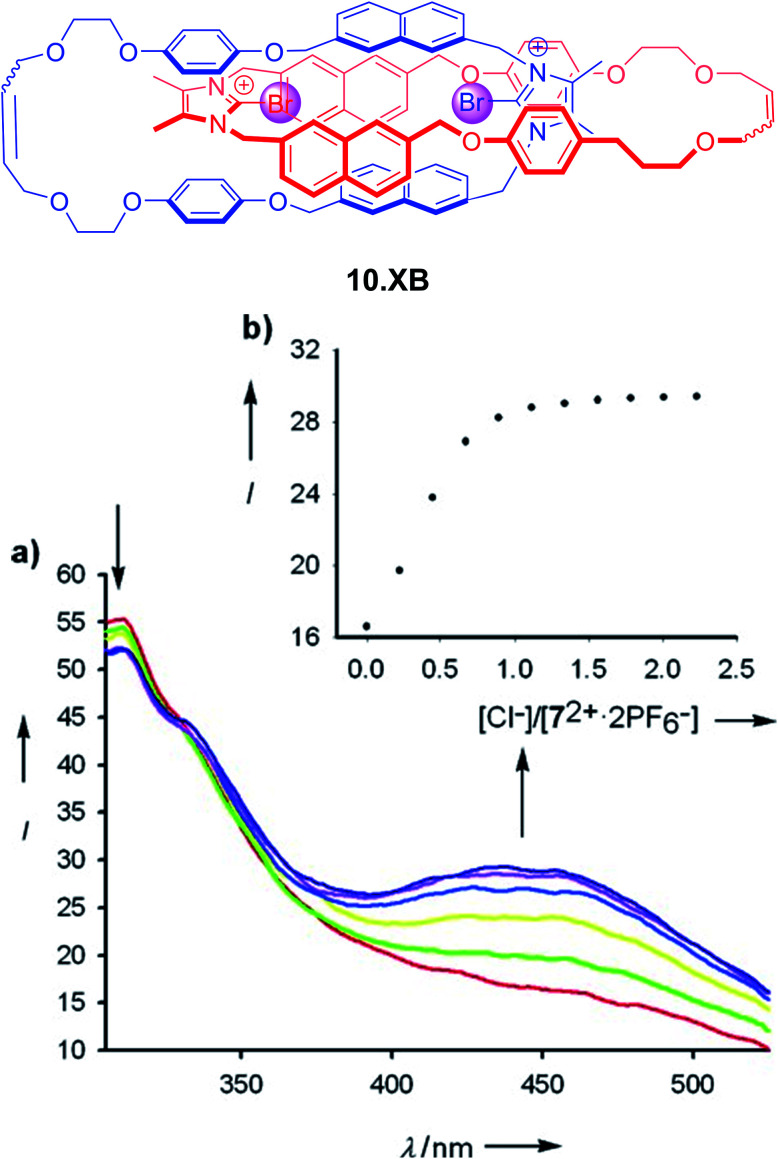
All-XB bromo-imidazolium-napthalene catenane 10.XB for fluorescent halide sensing. (a) Emission spectra of 10 μM 10.XB upon addition of Cl^−^ in ACN. (b) The corresponding response isotherm at 445 nm. Reproduced with permission from ref. 122 copyright 2011 Wiley.

The first example of a fluorescent XB interlocked host, and, to the best of our knowledge, the first example of XB-mediated fluorescent sensing in general, was reported by Caballero *et al.* in early 2012.^122^ In ACN the bis-bromo-imidazolium [2]catenane 10.XB, containing naphthalene reporter groups, displayed selective fluorescence switch-on only in the presence of Cl^−^ or Br^−^, with strong binding of 3.71 × 10^6^ M^−1^ and 148 000 M^−1^, respectively (Fig. 13). In contrast, a large range of other anions, including F^−^, I^−^, AcO^−^, H_2_PO_4_^−^, NO_3_^−^ and HCO_3_^−^ did not induce any fluorescence response. Similarly, the monomeric macrocyclic host precursor did not respond to any anions, highlighting the unique selectivity imbued by the preorganized, interlocked catenane XB binding cavity.

Another structurally related hetero-[2]catenane containing one XB iodo-triazolium macrocycle as well as a HB isophthalamide macrocycle component, was reported for fluorescent sensing of halides and oxoanions in ACN.^123^ All tested anions induced emission enhancements of the naphthalene emission, which were largest for the oxoanions AcO^−^ and H_2_PO_4_^−^ (+73 and +58% intensity increase in the presence of 20 equiv. anion). The response towards the halides was notably smaller with +29, +13 and +4% emission modulation for Cl^−^, Br^−^ and I^−^, respectively, thereby displaying a contrasting oxoanion selectivity in comparison to the bromo-imidazolium [2]catenane sensor 10.XB.

Fluorescent reporter motifs have also been incorporated into various rotaxane receptors. This includes, for example, a XB tris(iodo-triazole) rotaxane containing an anthracene reporter appended to the rotaxanes' macrocycle, capable of Cl^−^ sensing in ACN.^124^

**Fig. 14:**
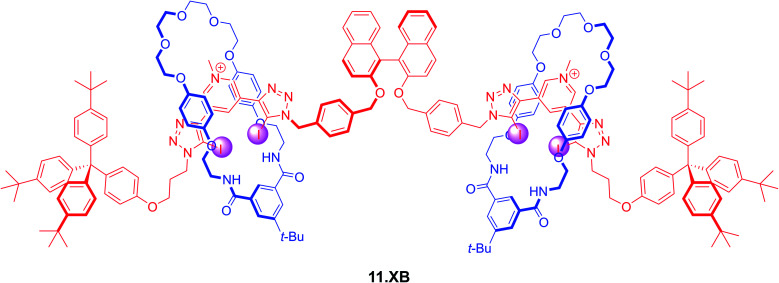
*S*-BINOL-based fluorescent [3]rotaxane 11.XB for fluorescent discrimination of *S*/*R*-glutamate enantiomers and fumarate/maleate geometric dicarboxylate isomers in CHCl_3_/MeOH/H_2_O 60 : 39 : 1.

Lim *et al.* also prepared a chiral XB [3]rotaxane fluorescent sensor 11.XB for biologically relevant dicarboxylates (Fig. 14).^125^^1^H NMR titrations in CDCl_3_/CD_3_OD/D_2_O 60 : 39 : 1 revealed significantly different binding modes between the rotaxane host and chloride and the dicarboxylates *S*-glutamate, *R*-glutamate, fumarate and maleate. While Cl^−^ was bound in a 1 : 2 host–guest stoichiometry with the halide binding within each individual interlocked cavity, dicarboxylate binding proceeded in a 1 : 1 fashion *via* formation of sandwich-type complexes.

From these ^1^H NMR studies significant Cl^−^ binding (*K*_1:1_ = 2610 M^−1^) was ascertained, while the chemical shift perturbations in the presence of the dicarboxylate guests were too small to be reliably analysed. However, fluorescence sensing studies in the same solvent system revealed strong dicarboxylate recognition with K of up to 35 200 M^−1^ for *S*-glutamate, as reflected in almost complete BINOL fluorescence quenching. Impressively, binding of the *R*-glutamate enantiomer was 5.7-fold attenuated, attesting to the unique potential of chiral interlocked hosts and sensors; the axle alone not only bound both guests much more weakly (*K* ≈ 1600 M^−1^) but also displayed no significant degree of enantiodiscrimination.

Similarly, the [3]rotaxane sensor displayed a significant preference for the more extended fumarate (*K* = 18 400 M^−1^), with significantly weaker binding of the maleate geometric isomer (*K* = 4180 M^−1^).

**Fig. 15:**
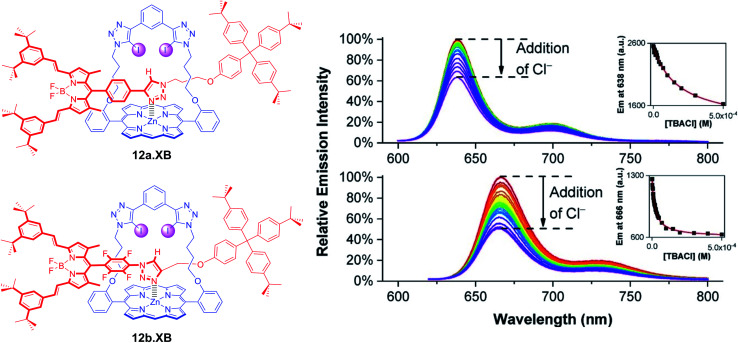
Fluorescence response of 1 μM XB BODIPY-containing strapped porphyrin rotaxanes 12a.XB (top) and 12b.XB (bottom) towards Cl^−^ in acetone. The insets show the corresponding response isotherms, highlighting stronger binding and a larger response for the more polarised, perfluorobenzene-containing 12b.XB. Reproduced from ref. 79 under the terms of the CC BY license.

A range of XB strapped-porphyrin receptors including BODIPY-containing rotaxanes 12a–b.XB were recently reported by Tse *et al.* (Fig. 15).^79^ The XB strapped-porphyrin macrocycle alone displayed significant changes (red-shift) in its Soret absorption band upon titration with halides in acetone, revealing strong anion binding which was enhanced up to 10 000-fold in comparison to the unfunctionalized parent Zn-tetraphenylporphyrin. This XB macrocyclic component was then integrated into a range of [2]rotaxanes, which showed significantly enhanced halide binding affinities in comparison to an analogous porphyrin-free rotaxane in d_6_-acetone and d_6_-acetone/D_2_O 98 : 2 as elucidated by ^1^H NMR studies. This can be attributed to an enhanced preorganisation and polarization of the interlocked binding cavity by axle-triazole Zn-porphyrin coordination. Unfortunately, this negated the ability of the metallo-porphyrin to act as a chromophoric reporting group; even in the presence of a >1000-fold excess of halides no colorimetric changes were observed. In order to restore the sensing capabilities of the rotaxanes, fluorescent BODIPY reporter groups were incorporated as axle components into 12a–b.XB. In acetone, both rotaxanes responded to Cl^−^, Br^−^, I^−^, OAc^−^ and SO_4_^2−^*via* BODIPY fluorescence quenching, whereby binding strength (and response magnitude) were larger for the more polarized perfluorophenyl-containing 12b.XB for all anions, as representatively shown for Cl^−^ in Fig. 15. In the presence of 2% water in acetone, only 12b.XB responded to Cl^−^ and Br^−^ with *K* = 1090 and 650 M^−1^, respectively, while 12a.XB did not respond to any anion. By virtue of the redox-activity of the Zn-porphyrin motif, the rotaxanes were also investigated as voltammetric anion sensors. In DCM, all rotaxanes displayed large cathodic voltammetric perturbations in the presence of HSO_4_^−^, OAc^−^ and Cl^−^, of up to −222 and −252 mV for OAc^−^ and Cl^−^ with 12a.XB, respectively. These XB rotaxanes represent rare examples of dual optical and electrochemical sigma–hole-mediated sensing.

**Fig. 16:**
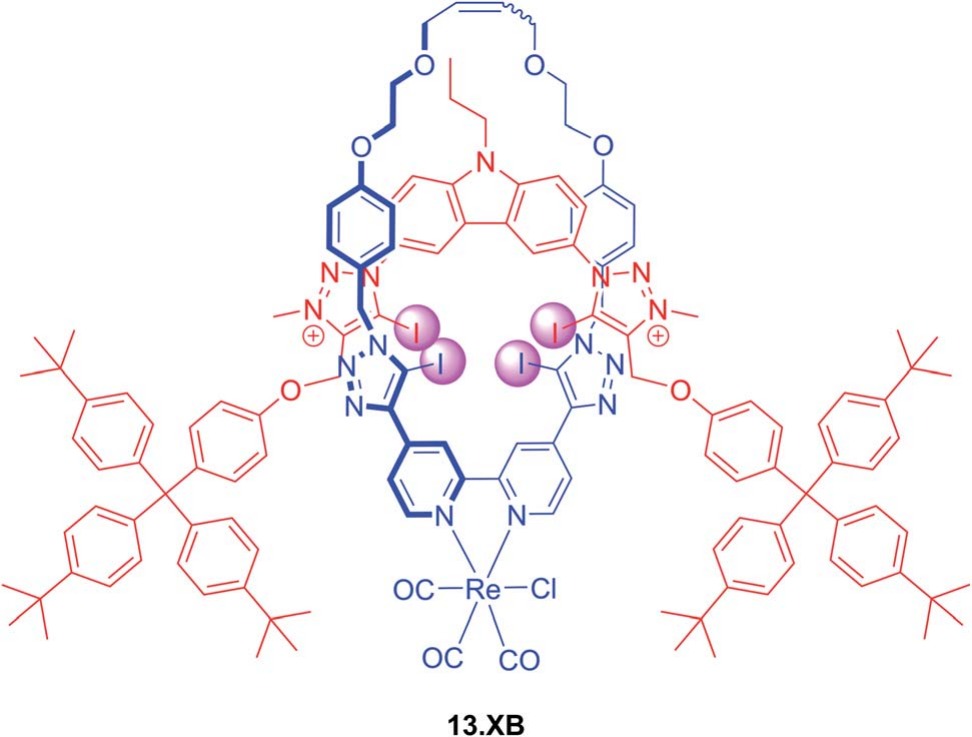
All XB Re(i)(CO)_3_Cl-containing [2]rotaxane 13.XB for selective fluorescent halide sensing in ACN/H_2_O mixtures.

The incorporation of transition metal-based luminescent reporters into interlocked XB hosts has also been investigated by Beer and co-workers, including the all-halogen bonding rotaxane 13.XB (Fig. 16).^126^ In ACN containing 10 or 20% H_2_O this Re(i)(bipy)-containing receptor displayed emission quenching in the presence of Cl^−^, Br^−^ and I^−^ while various oxoanions only induced minor or negligible perturbations. Halide recognition proceeded with 1 : 2 host–guest stoichiometry in both solvents with *K*_1:1_ of up to 138 000 M^−1^ for iodide, while Cl^−^ and Br^−^ were bound weaker and F^−^ did not bind at all. Even in ACN/H_2_O 1 : 1 selective halide fluorescence quenching was still observed, following the same binding trend (I^−^ > Br^−^ > Cl^−^) with *K*_1:1_ of up to 24 000 M^−1^.

A similar Re(i)(CO)_3_Cl-bistriazole rotaxane was also developed, which was however not luminescent.^127^ Langton *et al.* also integrated a Ru(bpy)_3_^2+^ reporter motif into a water-soluble XB rotaxane and demonstrated Br^−^, I^−^ and SO_4_^2−^ sensing in pure water, albeit with modest emission enhancements in the presence of excess anion of 3, 6 and 20%, respectively.^128^ In spite of a larger maximum response for sulfate, the halide anions were bound more strongly as their maximum emission response was reached at a concentration of 1 mM, while a higher concentration of 8 mM SO_4_^2−^ was required to induce signal saturation.

#### 3.2.3 Chalcogen bonding luminescent sensors

**Fig. 17:**
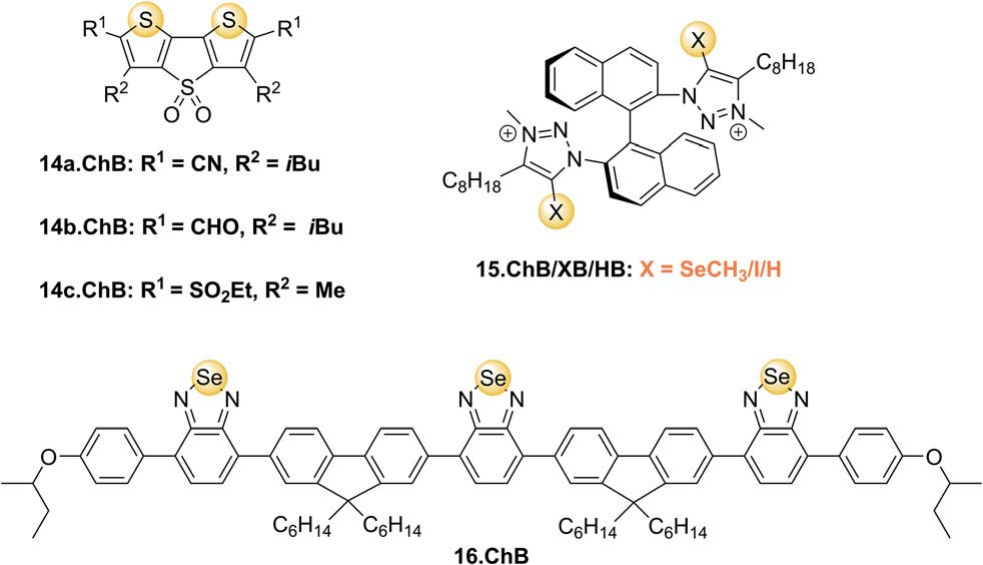
ChB fluorescent anion sensors.

In comparison to the numerous examples of luminescent XB sensors, the exploitation of ChB in optical sensing remains very rare. To the best of our knowledge, the first examples of emissive ChB sensors 14a–c.ChB (Fig. 17) were reported by the group of Matile in 2016.^36^ These dithienothiophenes (DTTs) have emerged as powerful (supra)molecular scaffolds with numerous applications, in particular as fluorescent probes.^129^ Particularly notable in the context of this review is their surprisingly potent ChB donor capability arising from convergently arranged sulfur-donor atom based σ–holes, polarized through the sulfone backbone.

This has been exploited in catalysis,^1^ anion binding and, in the afore-mentioned seminal work, for anion transport.^36^ Developed as transmembrane anionophores, DTT receptors 14a–c.ChB were also investigated as anion receptors and optical sensors. All three receptors displayed significant fluorescence emission quenching upon addition of up to 20 mM chloride in THF, with largest quenching and strongest binding of 885 M^−1^ and 204 M^−1^ observed for 14a.ChB and 14c.ChB, respectively. Both 14b.ChB (149 M^−1^) and a mono-cyano derivative of 14a.ChB (69 M^−1^) displayed weaker binding as well as smaller fluorescent responses. All three receptors also underwent small changes in absorbance in the presence of chloride. In contrast, as expected PF_6_^−^ did not induce any optical responses, while smaller fluorescence emission quenching of 14a.ChB and 14c.ChB was also reported in the presence of NO_3_^−^, in good agreement with weaker binding of this anion of 161 M^−1^ to 14a.ChB.

Beer and co-workers reported a series of chiral ChB/XB/HB (S)-BINOL based triazolium receptors 15.ChB/XB/HB for the recognition and fluorescent detection of stereo- and geometric dicarboxylate isomers in acetone/H_2_O 85 : 15.^130^ While 15.XB displayed significant chiral discrimination in the recognition of the enantiomers of tartrate and *N*-Boc-glutamate, both 15.HB and in particular 15.ChB did not display significant levels of enantioselectivity towards these chiral anion guests as elucidated by ^1^H NMR binding studies. In contrast, all receptors displayed a considerable degree of binding discrimination of the geometric isomers maleate/fumarate as well as phthalate/isophthalate with a preference for the more extended fumarate or isophthalate in all cases. For the former pair, 15.ChB displayed the largest discrimination with *K*_fum_/*K*_mal_ = 5.5, significantly better than 15.HB with *K*_fum_/*K*_mal_ = 2.0, while 15.XB decomposed upon exposure to malate.

**Fig. 18:**
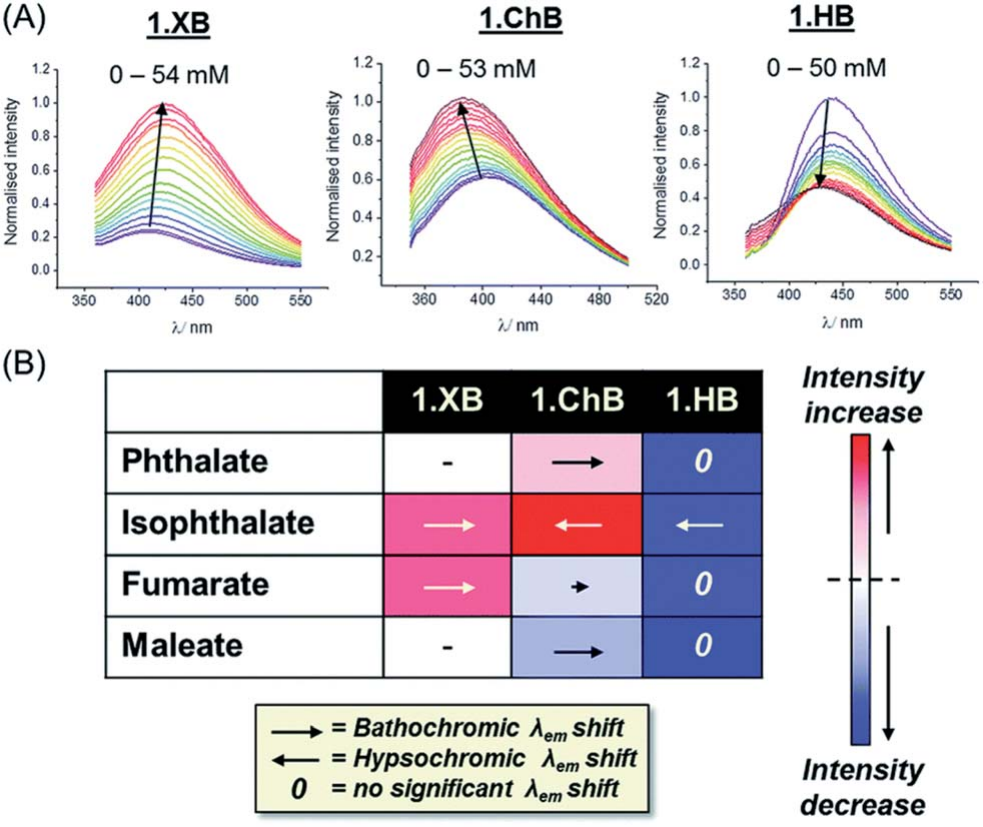
(A) Fluorescence emission spectra of 15.ChB/XB/HB (labelled 1.ChB/XB/HB in the figure) in the presence of increasing concentrations of isophthalate in acetone/H_2_O 85 : 15. (B) Summary of fluorescence response patterns of each receptor towards geometric dicarboxylate isomers. The length of the arrows is indicative of the magnitude of the shift in *λ*_max_ while the shade of red/blue denotes the magnitude of fluorescence enhancement/quenching. Reproduced from ref. 130 with permission from the Royal Society of Chemistry.

Similarly, both sigma–hole hosts displayed enhanced preference for isophthalate over phthalate in comparison to the HB congener. Interestingly, all three hosts displayed significantly different fluorescent anion sensing properties (Fig. 18).

While addition of all anions induced large emission enhancement for 15.XB, 15.HB showed quenching in all cases. In contrast, 15.ChB showed more nuanced response patterns with emission enhancements towards (iso)phthalate and quenching in the presence of fumarate/maleate. In addition, the ChB host also displayed unique changes in the emission wavelengths with opposite, hypso- and bathochromic shifts for isophthalate and phthalate, respectively.

**Fig. 19:**
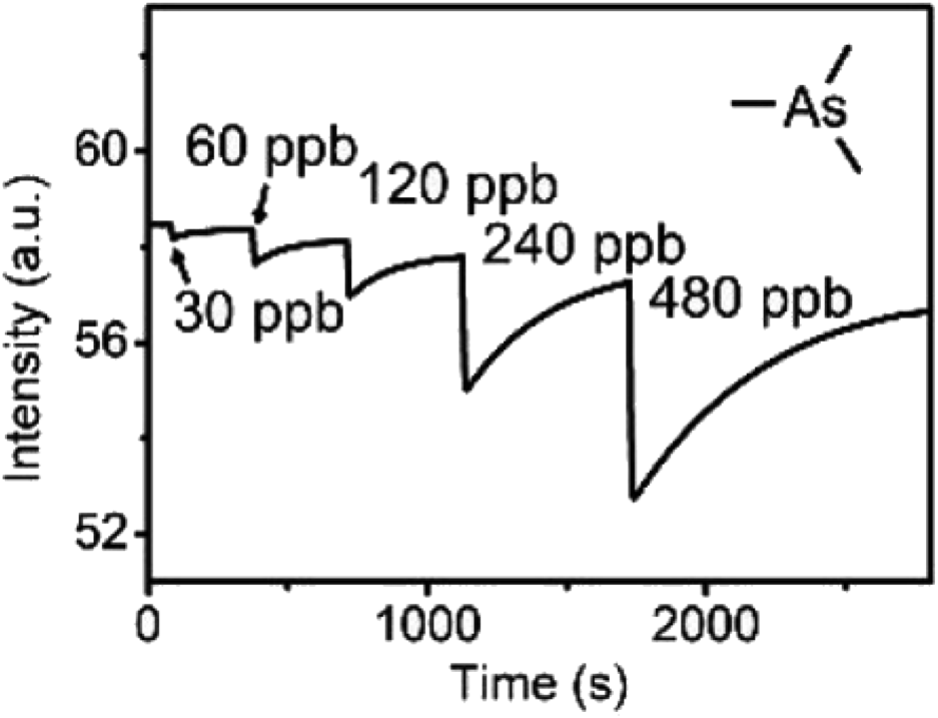
Fluorescence emission intensity of bundled nanoribbons of 16.ChB upon exposure to increasing concentration of trimethyl arsine gas. Reproduced with permission from ref. 131 copyright 2021 American Chemical Society.

Recently, Che and co-workers reported a fluorescent ChB sensor for the detection of the toxic trimethylarsine gas.^131^ This was achieved by formation of bundled nanofibers constructed from the tris(benzoselenadiazole) receptor 16.ChB which responded to exposure of the analyte vapours by a decrease in emission intensity (Fig. 19). With a LOD of 0.44 ppb and a fast response time of ≈3 s, this sensor performed significantly better than its sulfur-donor benzothiadiazole analogue (LOD = 67 ppb, response time of 54 s). This is indicative of the response arising from ChB-mediation recognition, as further supported by DFT calculations. The sensor displayed an impressive level of selectivity, with no notable response to a large range of solvent vapours, including H_2_O, methanol, ethanol and acetone at significantly higher levels. In addition, selective trimethylarsine detection was also demonstrated in complex matrices, including car exhaust, smoke and hair spray.

The same group also developed a structurally related ChB benzoselenadiazole sensor for assessment of meat freshness by fluorescent sensing of gaseous dimethyl sulfide.^132^

## 4. Electrochemical sensors

Owing to their low cost, high sensitivity, flexibility, and scalability, electrochemical sensors are at the forefront of sensor development,^133^ in particular for the sensing of biomolecules,^134^ but also for small molecules and ions.^40^ The latter most notably includes ion-selective electrodes, which, as alluded to in Section 2.1, are thus far the only widely and generically applied ion sensors. This can in part be attributed to a century-long development of potentiometric techniques, their low cost and (operational) simplicity.^46–49^ Nevertheless, these are not available for various (an)ions and, depending on the specific application scenario, can fail to address certain sensing criteria (*e.g.* selectivity, or the capability to monitor small changes in concentration), see also Section 4.2.§§Note that the selectivity of ISEs is in principle governed by the supramolecular design of the ionophore hosts. However, in comparison to other sensing approaches the selectivity is further affected by the inherent partitioning of ions into the ISE membrane. Hence, ions of lower hydration enthalpy often interfere with membrane based ISEs. This has sparked significant research into alternative electroanalytical supramolecular host–guest ion sensing methodologies, in particular voltammetric sensors based on redox-active receptors as discussed in the following Section.

### 4.1 Redox-active sensors

#### 4.1.1 Solution-phase voltammetric anion sensing

**Fig. 20:**
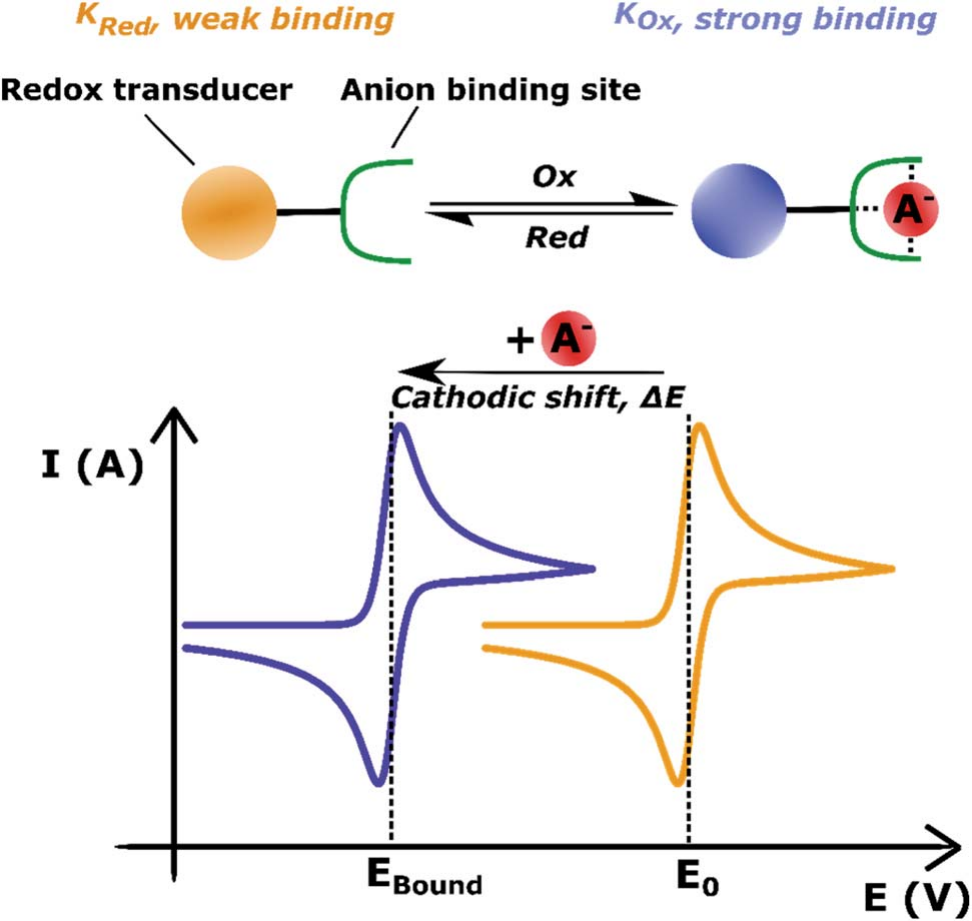
Schematic depiction of voltammetric anion sensing at a redox active anion receptor. Receptor oxidation/reduction is reflected in anion binding enhancement/decrease. In the presence of a binding anion the half-wave potential is perturbed cathodically, whereby the shift magnitude is directly proportional to the ratio of anion binding constants to the different receptor oxidation states: Δ*E* ∝ *K*_Ox_/*K*_Red_.

The integration of redox-active reporter groups, in particular ferrocene (Fc), into synthetic receptors is a well-established approach to generate potent sensors whose voltammetric properties are dependent on the presence of the bound guest species.^40,71^ Specifically, recognition of a Lewis basic (typically anionic) guest enhances the electron density at the redox active receptor, stabilising the higher oxidation state, which is reflected in a cathodic voltammetric perturbation (shift to more negative potentials, easier oxidation) of the redox couple. This change in the receptor's half-wave potential (*E*_1/2_) is then readily measurable by simple voltammetric techniques such as cyclic voltammetry (CV), differential pulse voltammetry (DVP) or square-wave voltammetry (SWV), see Fig. 20.

In turn, voltammetric modulation of the receptor's redox state affects the guest binding properties, with stronger anion binding in the higher, more cationic oxidation state (*K*_Ox_) than in the lower, neutral or anionic redox state (*K*_Red_). The specific signalling pathways and fundamentals that underpin these observations are well-established in the literature, but will not be discussed in detail herein.^40,135–138^ However, note that in the most general case the magnitude of the voltammetric perturbation is given by 
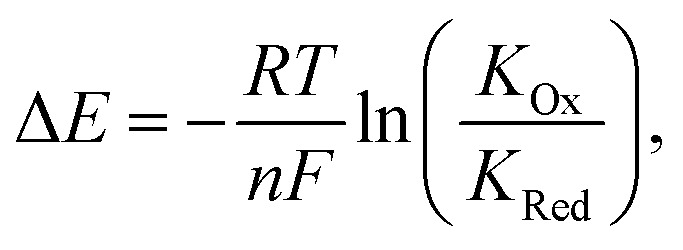
 and is thus only dependent on the ratio of *K*_Ox_/*K*_Red_ (often denoted the binding enhancement factor, BEF).^40^ This is to say that the signal magnitude in a voltammetric (an)ion sensor is determined by how strongly guest binding is affected by receptor oxidation/reduction. From this consideration it becomes apparent that a stronger electronic communication between the redox and receptive sites is a key factor for sensor performance. It is thus not surprising that sigma hole interactions, which, as discussed in Section 2.2, display a high degree of electronic tuneability, are particularly potent in voltammetric (anion) sensors.^139^

The first demonstration of a high sensitivity of redox control of XB was reported by Schöllhorn and co-workers in 2014, wherein it was shown that the voltammetric properties of redox active Lewis bases can be modulated in the presence of neutral XB donors (note that this is the “reverse” case as depicted in Fig. 20 and in subsequent examples).^140^ For instance, in ACN the reduction of tetrachloro-*p*-quinone (TCQ) is facilitated in the presence of iodo-perfluoro-alkynes/arynes. Specifically, CV experiments showed that single-electron reduction of TCQ to TCQ^−^˙ is not perturbed by addition of 1-iodo-perfluorohexane, while the second reductive couple TCQ^−^˙/TCQ^2−^ undergoes significant anodic voltammetric shifts of up to 140 mV in the presence of up to 100 equivalents of XB donor. This is indicative of XB formation, and concomitant decrease in electron-density, which occurs only for the stronger, dianionic Lewis base TCQ^2−^.

**Fig. 21:**
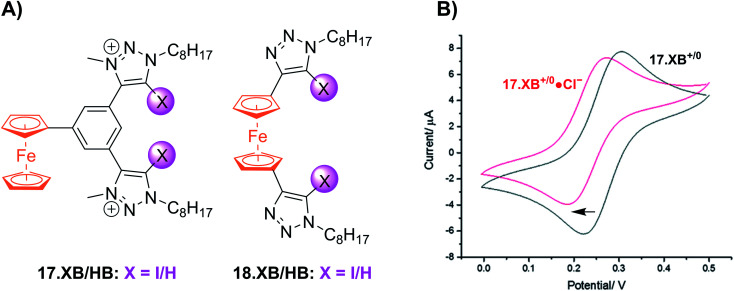
(A) Chemical structures of the first XB ferrocenyl voltammetric anion sensors. (B) CVs of 17.XB in ACN in the absence (black) and presence of 10 equiv. Cl^−^ (red). Adapted from ref. 141 with permission from the Royal Society of Chemistry.

Shortly thereafter the Beer group reported the first examples of redox active XB iodotriazole voltammetric anion sensors 17.XB and 18.XB (Fig. 21A).^141^ In this case, and all following examples, the XB receptor itself is redox-active, such that the XB donor strength is electrochemically modulated and sensing of redox-inactive Lewis basic analytes, in particular anions, is enabled. In ACN both receptors responded to presence of up to 10 equiv. of Cl^−^ or Br^−^ with moderate cathodic shifts of ≈−30 and ≈−20 mV, respectively (Fig. 21B), which is notably larger than the response of their HB congeners 17.HB and 18.HB. In particular the response of 18.HB was strongly diminished with −6 and 0 mV for Cl^−^ and Br^−^, respectively, highlighting the crucial role of the XB interaction in sensing the halide anions.

**Fig. 22:**
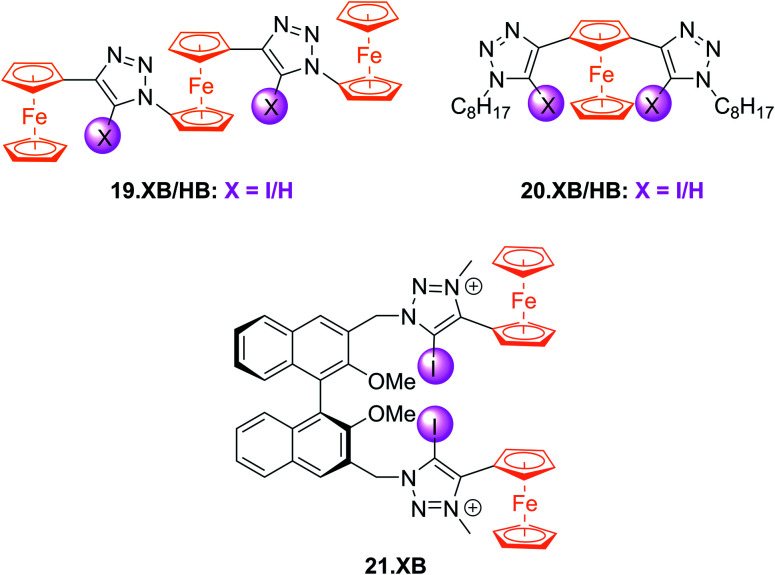
Chemical structures of Fc-based XB and HB voltammetric sensors for oxoanions, azide and chiral anions.

Subsequently, a range of XB Fc-containing acyclic receptors were prepared by different groups. For example, Zapata, Caballero and Molina reported trisferrocene-bis((iodo)triazole) receptors 19.XB/HB (Fig. 22) as oxoanion sensors in DCM/ACN 1 : 1.^142^ Notably, the receptors display two redox waves as a result of strong electronic coupling between the two chemically inequivalent Fc environments, whereby the outer Fc motifs are simultaneously oxidised first, while the inner Fc is subsequently oxidised at a ≈400 mV higher potential. In addition, the sensors display comparably complex voltammetric response patterns, characterised by a two-wave slow exchange behaviour and response magnitudes that differ significantly for both redox waves. For example, addition of OAc^−^ or SO_4_^2−^ to 19.XB did not perturb the first, more cathodic redox wave, but induced moderate perturbations of the second redox couple of −65 and −52 mV, respectively. In contrast, in the presence of H_2_PO_4_^−^ or HP_2_O_7_^3−^ both redox couples were cathodically perturbed, in particular the more anodic wave, displaying shifts of −327 and −252 mV, respectively. Furthermore, and in contrast to all other solution-phase examples discussed herein, 19.XB shows somewhat smaller voltammetric shift perturbations than its HB analogue. These observations may arise as a result of the dominance of strong coulombic electrostatic interactions between the anions and the di/tricationic receptors in the low polarity solvent system.

Lim *et al.* further developed the voltammetric Fc-based sensors 20.XB/HB and 21.XB. The former, a constitutional isomer of the afore-discussed 18.XB/HB was developed for sensing of azide in ACN/H_2_O 99 : 1.^143^ Receptor 20.XB notably displayed larger responses towards this anion of −40 mV over Cl^−^, Br^−^ or OAc^−^ (max. −22 mV for Br^−^, all at 10 equiv.) as well as significantly enhanced signal magnitudes in comparison to 20.HB (−18 mV for N_3_^−^).

The chiral 21.XB represents a rare example of voltammetric enantioselective sensing of various chiral anions, achieved in ACN.^144^ This (*S*)-BINOL-based XB probe displayed a larger response for the *R*-enantiomer of both *N*-Boc-alanine and *N*-Boc-leucine with Δ*E*_R_/Δ*E*_S_ of 1.11 and 1.41, respectively and a preferential response towards the *S*-enantiomer of BINOL-phosphate with Δ*E*_R_/Δ*E*_S_ = 0.67. These voltammetric enantioselectivities are not only in very good agreement with those obtained by ^1^H NMR binding titrations of the neutral receptor but are also larger than those observed in a previous Fc-urea HB sensor.^145^ These observations highlight the potential of XB systems not only as potent voltammetric sensors with typically enhanced response magnitudes in comparison to HB analogues, but also enhanced enantiodiscrimination, presumably arising from the stricter geometric preferences imposed by XB.

**Fig. 23:**
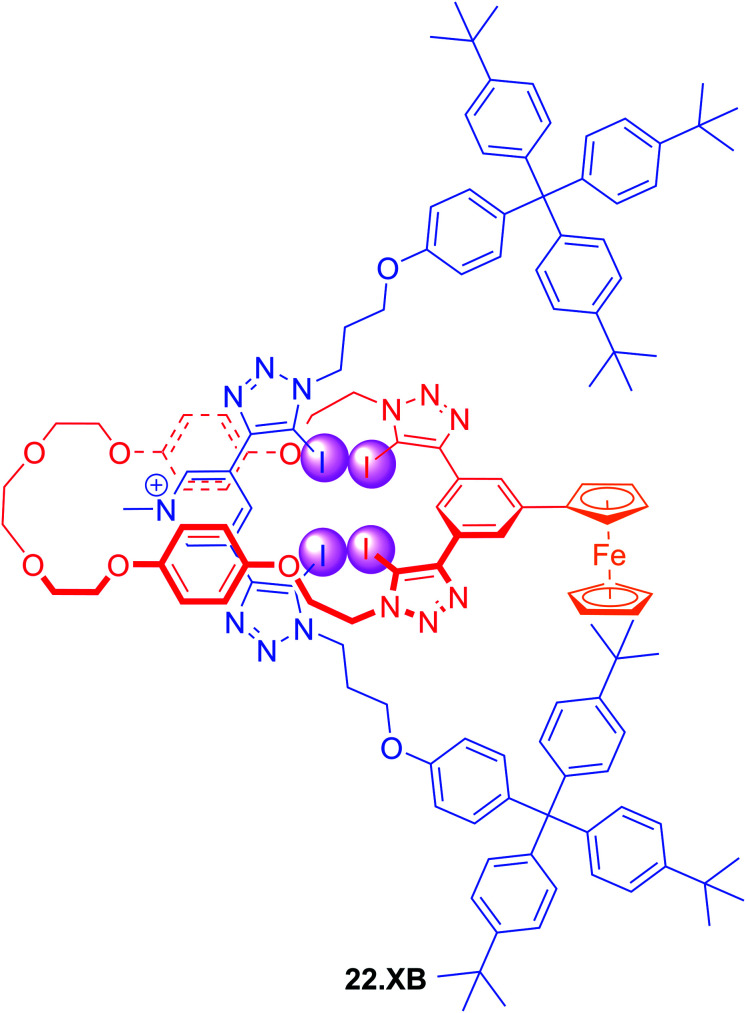
Ferrocene-containing all XB [2]rotaxane for selective voltammetric sensing of bromide in ACN/acetone/H_2_O 45 : 45 : 1.

In an effort to further enhance the selectivity of such voltammetric anion sensors, Lim and Beer recently integrated a Fc-reporter group into an all-XB rotaxane 22.XB.^146^ In the competitive solvent mixture of ACN/acetone/H_2_O 45 : 45 : 1, this sensor displayed a modest but notably selective response towards excess Br^−^ of −22 mV over Cl^−^ and SCN^−^. This selectivity is in good agreement with the binding preference of the native rotaxane as elucidated by ^1^H NMR binding titrations (Fig. 23).

**Fig. 24:**
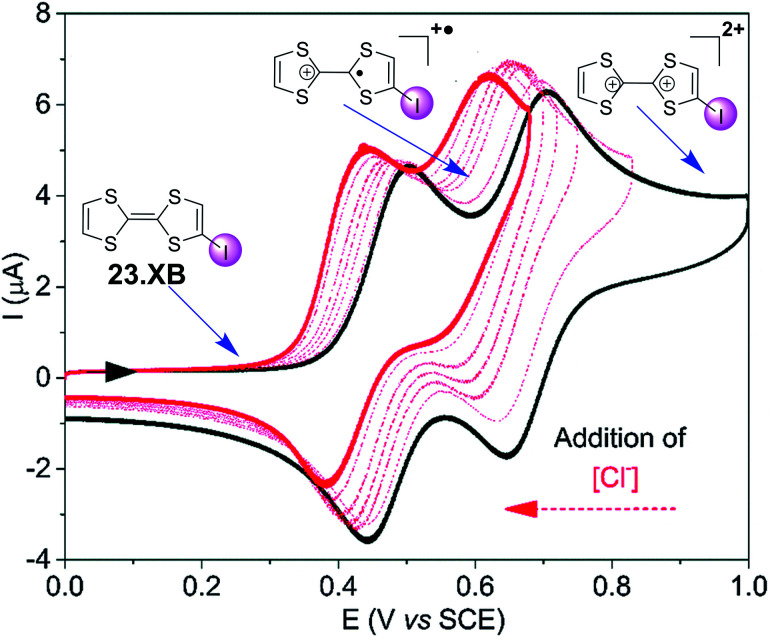
Chemical structure of iodo-TTF 23.XB and its corresponding CV in DMF in the absence (black) and presence (red) of increasing concentrations of Cl^−^. Depicted are also the different redox states at different potentials (structures and blue arrows). Adapted from ref. 138 with permission from the PCCP Owner Societies.

The groups of Beer as well as Schöllhorn and Fave also investigated a range of other redox transducers in XB anion sensors, such as viologen-based systems for detection of various halides, whereby all studies revealed an important contribution of XB in obtaining (enhanced) voltammetric responses.^147–149^ The latter groups further conducted a range of systematic studies into XB iodo-tetrathiafulvalene (TTF) voltammetric sensors.^138,150^ As shown in Fig. 24, iodo-TTF 23.XB displays two reversible oxidative couples in DMF, corresponding to step-wise one-electron oxidation to 23.XB^+^˙ and 23.XB^2+^, which both respond to the presence of increasing chloride concentrations by well-defined, continuous cathodic shifts. As expected, various control experiments proved that XB formation was the crucial driving force in halide recognition and sensing.^138,150^

**Fig. 25:**
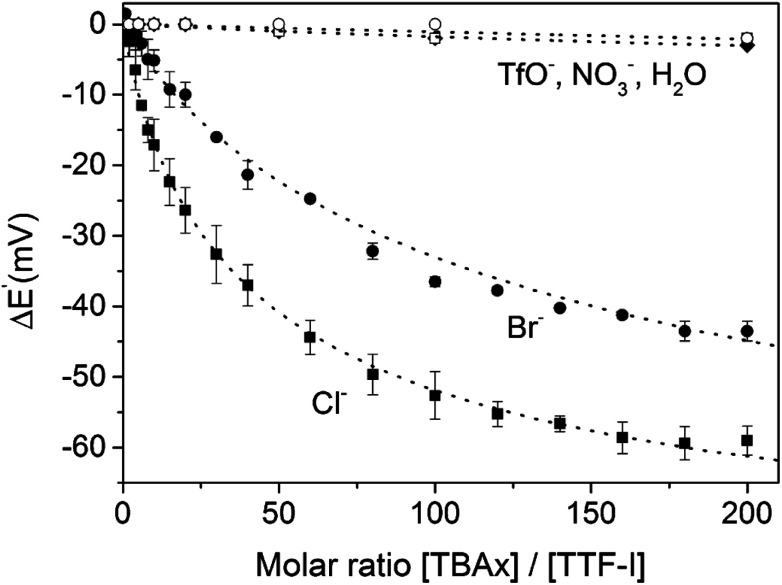
Cathodic voltammetric perturbations of the first oxidative couple of iodo-TTF 23.XB in DMF upon addition of various anions. Reproduced from ref. 138 with permission from the PCCP Owner Societies.

The sensor further displayed somewhat smaller cathodic perturbations in the presence of Br^−^, while OTf^−^, NO_3_^−^ and H_2_O did not induce any response (Fig. 25). By fitting of the voltammetric binding isotherms to a 1 : 1 host–guest stoichiometric Nernst binding model the authors further extracted absolute halide binding constants to all receptor oxidation states.^40,138^

Unsurprisingly, the neutral, native 23XB displayed only very weak halide anion binding constants (≤20 M^−1^), while binding to the monocationic 23XB^+^˙ was significantly switched on with *K* = 425 and 131 M^−1^ for Cl^−^ and Br^−^, respectively. A further increase in chloride binding to 23XB^2+^ of *K* = 6730 M^−1^ was extracted, while the analogous binding constant for Br^−^ could not be obtained as Br^−^ oxidation overlapped with the second oxidative TTF couple in CV. These observations saliently highlight the unique advantages of voltammetric anion sensors; the transient generation of a more cationic, higher oxidation state increases anion binding to such an extent that sensing in competitive solvent media, in which the native receptor often displays negligible anion binding, is possible. This concept was also recently exploited for the sensing of anions in competitive aqueous/organic solvent systems at a range of interfacial XB anion sensors, as discussed in more detail in Section 4.1.2.^57,71,72,137^

The same groups later reported a systematic investigation of rarely studied electrolyte effects on the anion sensing performance of a methylated iodo-TTF derivative of 23.XB.^150^ The authors showed that different electrolyte anions BF_4_^−^, MsO^−^, TfO^−^, NO_3_^−^, ClO_4_^−^, PF_6_^−^ or BAr^F^_4_^−^ ([tetrakis[3,5-bis(trifluoromethyl)phenyl]borate]) can significantly influence the sensing properties of (XB) voltammetric anion sensors. For instance, the cathodic shift perturbation of XB sensor trimethyl-iodo-TTF towards 100 equiv. Cl^−^ ranged between −36 and −49 mV, corresponding to an up to 2.6-fold difference in *K*_Ox_, depending on the electrolyte. These observations highlight that even “non-coordinating” electrolyte anions may significantly compete with anion binding and associated signal transduction in redox-active sensors. This was recently corroborated by a systematic comparison of NMR, UV-vis and voltammetrically determined anion binding constants in XB viologen derivatives in the absence and presence of electrolytes.^149^

**Fig. 26:**
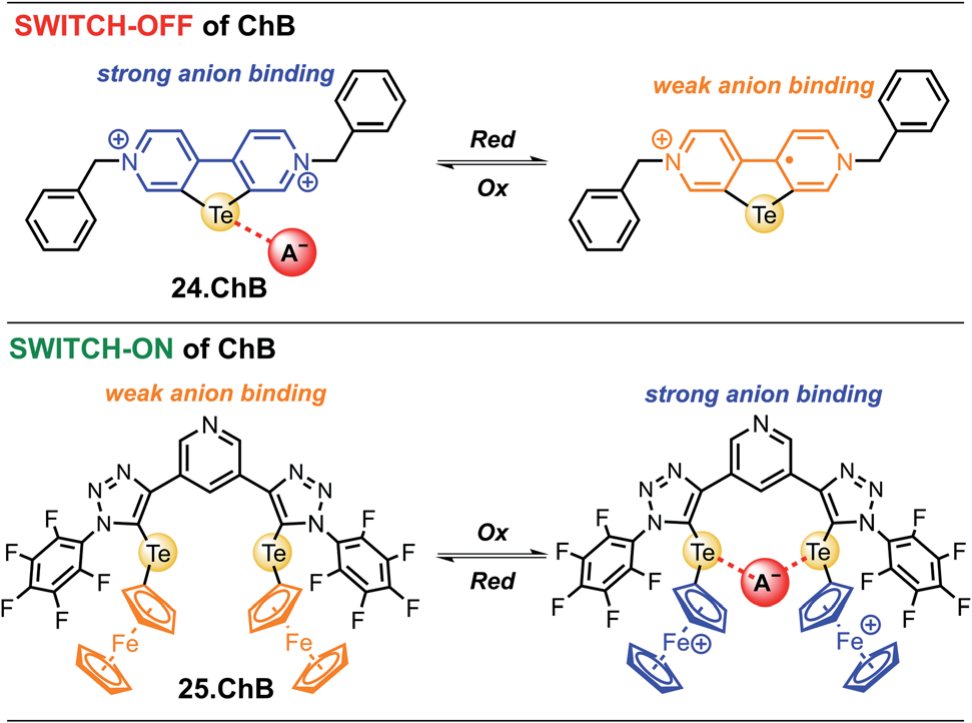
ChB-mediated voltammetric anion sensing was very recently achieved for the first time as demonstrated for both reductive switch-OFF of ChB in telluroviologen 24.ChB as well as oxidative switch-ON of ChB in pyridine bis(ferrocenyltelluroviologen) 25.ChB.

In 2022, Hein *et al.* reported the first examples of ChB voltammetric anion sensors including the dicationic telluro-viologen derivative 24.ChB as well as the neutral pyridine bis(ferrocenyltellurotriazole) 25.ChB (Fig. 26).^62^

**Fig. 27:**
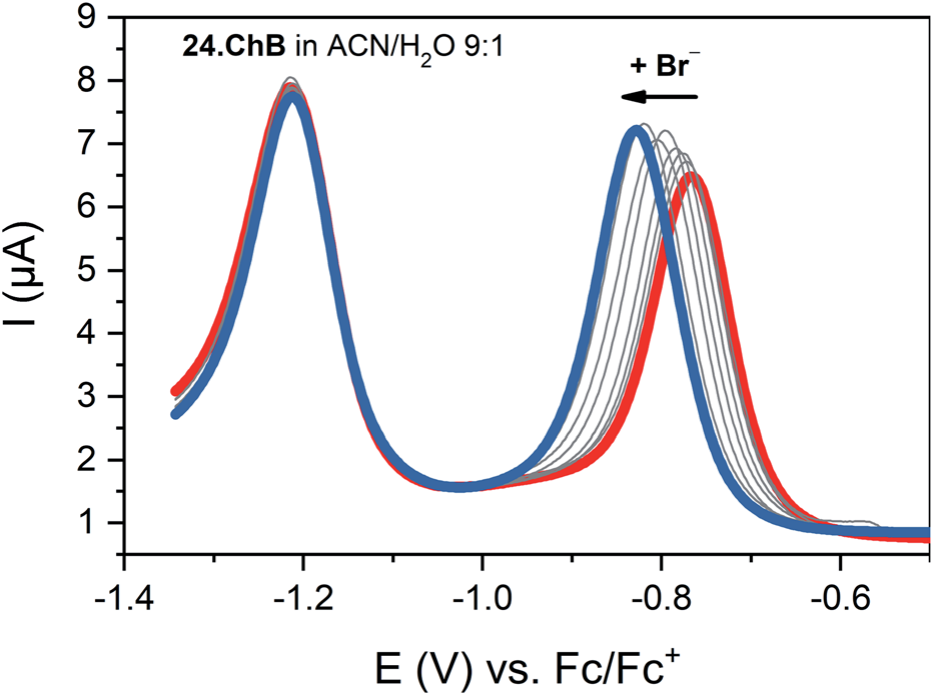
SWVs of telluroviologen ChB sensor 24.ChB in ACN/H_2_O 9 : 1 upon addition of up to 50 mM Br^−^. Only the first reductive couple is responsive, indicative of complete ChB deactivation upon mono-reduction. Reproduced with permission from ref. 62 copyright 2022 American Chemical Society.

The telluro-viologen 24.ChB displayed moderately strong halide binding in competitive CD_3_CN/D_2_O 9 : 1 with a modest preference for bromide (*K* = 1036 M^−1^) while its lighter Se congener bound all halides much more weakly (*K* = 182 M^−1^ for Br^−^), yet still stronger than the HB viologen analogue (*K* = 139 M^−1^), confirming a significant ChB participation in anion recognition. Voltammetric anion sensing studies in the same solvent system confirmed significant cathodic responses of the first reductive viologen couple of the telluoroviologen 24.ChB, which were again largest for bromide (Δ*E*_max_ = −61 mV), and slightly smaller for chloride (−57 mV) and iodide (−49 mV). In contrast, the oxoanions HSO_4_^−^ and NO_3_^−^ induced smaller responses of ≤−36 mV. These perturbations were only observed for the first reductive couple; the second reduction couple was not perturbed in the presence of any anion, see Fig. 27. This indicates that upon first mono-electron reduction, the receptor's potent ChB ability is switched-OFF *i.e.* the bound anion is expelled. A further reduction has thus no additional effect on anion binding and no shifts of the second couple are observed. Importantly, both the lighter ChB seleno-congener as well as the unfunctionalized HB viologen responded to anions much more weakly, with largest responses towards Cl^−^ of −22 and −17 mV, respectively. Of further note is that 24.ChB also responded to the halides *via* naked eye-visible changes in absorbance (red-shift), thereby acting as a dual-output anion sensor.

In contrast to the reductive switch-OFF telluro-viologen system, the neutral ferrocenyltellurotriazole receptor 25.ChB was investigated as a ChB switch-ON sensor *via* ferrocene oxidation.

In its natively neutral state 25.ChB displayed very low ChB potency as elucidated by ^1^H NMR anion binding studies; even in the less competitive acetone only H_2_PO_4_^−^ displayed modest binding of 111 M^−1^.

Nevertheless, the sensor displayed large cathodic perturbations of the ferrocene/ferrocenium redox couple upon addition of various halides (Cl^−^, Br^−^) as well as oxoanions (H_2_PO_4_^−^, HSO_4_^−^, NO_3_^−^), in a range of competitive solvent systems, including ACN, ACN/H_2_O 19 : 1 and ACN/H_2_O 9 : 1, attesting to the strong ChB switch-ON upon receptor oxidation. In ACN, 25.ChB responded with the following selectivity trend: H_2_PO_4_^−^ > Cl^−^ > Br^−^ > HSO_4_^−^ > NO_3_^−^, with perturbations of up to −217 mV for H_2_PO_4_^−^. This corresponds to an impressively large binding-enhancement factor BEF = 4800. Even in the presence of 10% water in ACN the sensor still displayed cathodic voltametric shifts of up to −42 mV towards bromide and a notably altered selectivity trend: Br^−^ > H_2_PO_4_^−^ ≈ Cl^−^ > HSO_4_^−^ ≈ NO_3_^−^. In comparison to other similar XB/HB voltammetric sensors, 25.ChB displays in general significantly enhanced Br^−^ responses, which are up to 2.4-fold larger than those observed for structurally similar 27.XB in both ACN and ACN/H_2_O 19 : 1.^57^ This confirms a particularly potent redox-dependent binding-modulation of ChB (large BEF and large Δ*E*), enabled by a high sensitivity of ChB on its electronic environment^28^ as well as the uniquely close spatial coupling of the redox and Te-donor binding sites, a design principle with significant future potential. Importantly, these findings establish redox-control of ChB as a powerful, reversible approach for high fidelity switch-OFF or switch-ON modulation of ChB anion recognition and sensing.

#### 4.1.2 Interfacial redox-active anion sensors

**Fig. 28:**
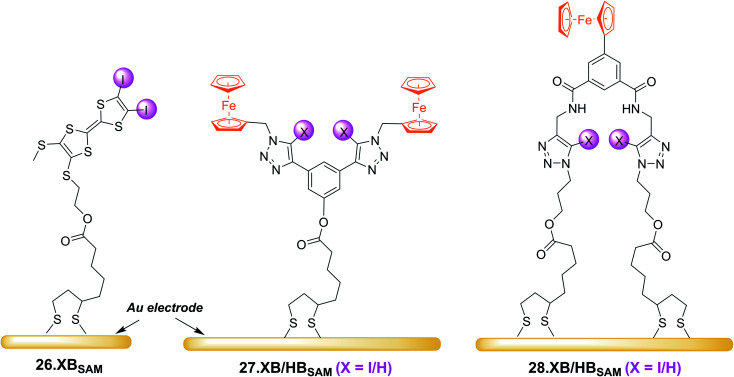
Schematic depiction of recently developed XB redox active self-assembled monolayers on gold electrodes. Note that for comparative solution-phase studies chemically related derivatives of 27.XB/HB and 28.XB/HB were also studied under diffusive conditions.

In another recent development, redox-active XB receptors were immobilised onto electrode-surfaces to furnish surface-confined anion sensors. This is associated with numerous advantages over solution-phase sensing, most importantly enhanced sensory responses, circumventing solubility constraints, facile device integration, potential for sensor reuse and sensing under flow.^40,151^ The first example of such an interfacial XB voltammetric sensor, the self-assembled monolayer (SAM) of a bisiodo-TTF derivative 26.XB_SAM_ (Fig. 28), was reported in 2019 by Fave, Schöllhorn and co-workers.^152^ In ACN, this interface voltammetrically responded to the halides Cl^−^ and Br^−^, with cathodic perturbation of the first TTF oxidative redox couple of ≈ −150 mV towards Cl^−^. Of particular note is that this behaviour differs distinctly from that of the same receptor studied in solution. Under diffusive conditions, the halide response is not only smaller (up to ≈ −95 mV in presence of 200 equiv. Cl^−^), but is also characterised by continuous cathodic shifts (as also observed for the related 23.XB, see Fig. 24 and 25), however these studies were, for solubility reasons, carried out in a different solvent system of ACN/DMF 3 : 7. In contrast, the response of 26.XB_SAM_ follows a more complex slow-exchange two-wave pattern with emergence of a new peak at lower potentials, a result of altered kinetic binding profiles. These results nicely illustrate the afore-mentioned advantages of interfacial sensing, that is circumvented solubility constraints and improved response magnitudes. The latter was justified by elucidation of the Cl^−^ binding constants to 26.XB_SAM_ and 26.XB_SAM_^+^˙, which were with *K*_Red_ ≈ 1000 M^−1^ and *K*_Ox_ ≈ 570.000 M^−1^, not only individually larger than those in solution, but whose ratio (*i.e.* the BEF) was also significantly enhanced, corresponding to a larger response magnitude (Δ*E* ∝ *K*_Ox_/*K*_Red_).

An enhanced interfacial response of the bis(ferrocene-(iodo)-triazole) sensors 27.XB/HB_SAM_ towards the oxoanions HSO_4_^−^, H_2_PO_4_^−^ and NO_3_^−^ in various ACN/H_2_O mixtures of up to 30% water was also reported by Patrick *et al.* in 2021.^57^

Interestingly, the HB congener 27.HB_SAM_ displayed a slightly enhanced response towards these oxoanions in comparison to 27.XB_SAM_, while under diffusive conditions 27.XB_dif_ outperformed 27.HB_dif_ in response to all anions, including Cl^−^ and Br^−^, in a range of ACN/H_2_O mixtures of up to 20% water. This unexpected observation potentially arises from differing interfacial receptor organizational or hydration differences as elucidated by various surface analyses. Particularly noteworthy in this study is a rare demonstration of XB/HB (interfacial) voltammetric sensing in highly competitive aqueous media of up to 30% water (in which the diffusive receptors are not soluble), again highlighting the utility of surface-immobilisation in generating potent, potentially real-life relevant electrochemical anion sensors. This work also presents the first comprehensive study into solvent effects in voltammetric anion sensors. Unsurprisingly, the voltammetric shift magnitude of both sensors, in solution and at the surface, generally decreased upon increasing water content, particularly strongly for H_2_PO_4_^−^, a reflection of its large hydration enthalpy. A noteworthy exception to this trend is the solution-phase performance of 27.XB_dif_, whose response to the halides was largely independent of water content (note that this effect could not be studied at the interfacial receptors due to poor voltammetric reversibility of 27.XB/HB_SAM_ in the presence of halides). Importantly, this trend was not observed for 27.HB_dif_; with increasing water content the anion sensing performance of 27.XB_dif_*relatively* increased in comparison to the HB sensor, attesting to the potency of XB anion sensing in aqueous media.

**Fig. 29:**
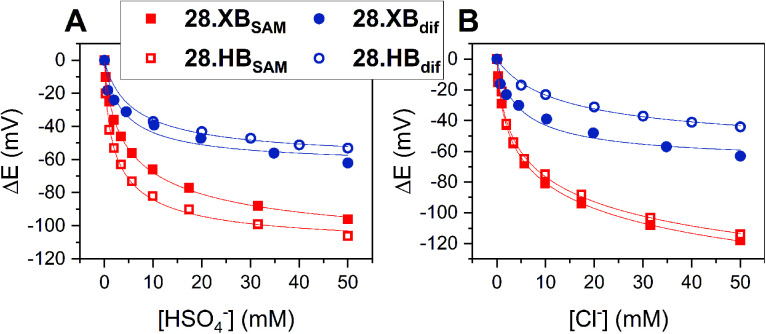
Comparison of cathodic voltammetric shifts under diffusive conditions of 28.XB/HB_dif_ (blue circles) and on the surface (28.XB/HB_SAM_ (red squares)) in ACN/H_2_O 99 : 1 upon titration with (A) HSO_4_^−^ and (B) Cl^−^. Filled symbol represent the XB receptors while empty symbols represent the HB receptors. A pronounced surface enhancement was also observed for all other tested anions (Br^−^, H_2_PO_4_^−^ and NO_3_^−^). Adapted from ref. 137 with permission from the Royal Society of Chemistry.

A detailed investigation into the transduction mechanisms that govern the response mechanisms and enhanced signal magnitudes of interfacial voltammetric sensors was recently reported by Hein *et al.*^137^ As shown in Fig. 29, the XB/HB ferrocene-isophthalamide-(iodo)triazole interface 28.XB/HB_SAM_ displayed significantly enhanced sensory responses towards a range of oxoanions as well as halides in ACN/H_2_O 99 : 1.

In analogy to the 27.XB/HB sensor system, the interfacial response towards the oxoanions HSO_4_^−^, H_2_PO_4_^−^ and NO_3_^−^ was slightly augmented for 28.HB_SAM_, while 28.XB_SAM_ displayed a modest preference towards the halides Cl^−^ and Br^−^, in agreement with previous observations of a typical XB recognition preference towards (softer) halides.^14,23^

In good agreement with the above-described voltammetric studies is also the consistently augmented solution-phase performance of 28.XB_dif_ towards all anions. All of these observations were rationalised in the context of a novel dielectric model, highlighting the importance of through-space and through-bond charge interactions and their screening in environments of different dielectric.

**Fig. 30:**
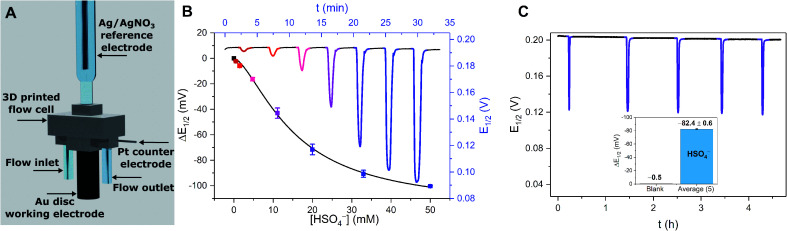
(A) Schematic depiction of the 3D-printed electrochemical flow cell. (B) Sensogram (blue axes) and corresponding isotherm (black axes) of 28.XB_SAM_ under continuous flow to increasing concentrations of HSO_4_^−^ in ACN/H_2_O 99 : 1 as measured by continuous SWV. Each spike corresponds to injections of increasing [HSO_4_^−^] up to 50 mM. (C) Voltammetric response of 28.XB_SAM_ towards five additions of 20 mM HSO_4_^−^ under continuous electrolyte flow over 4.5 h. The inset shows the voltammetric shift in response to the blank electrolyte and the average of all five HSO_4_^−^ additions. Reproduced from ref. 72 under the terms of the CC BY license.

As a result of its well-defined anion sensing performance and high voltammetric stability the groups of Beer and Davis further developed 28.XB/HB_SAM_ as anion sensors for real-time continuous flow sensing. The detection of anions in aqueous media under flow conditions is of high relevance across various applications, including long-term health and water monitoring, but remains underdeveloped. Interfacial supramolecular sensors are ideally suited to address this challenge as they can be easily re-used by simple washing. To demonstrate this capability, Patrick and Hein *et al.* developed a 3D-printed electrochemical flow cell (Fig. 30A) through which electrolyte was continuously pumped over a 28.XB/HB_SAM_-modified gold electrode.^72^

A continuous sensor signal readout (that is the *E*_1/2_) of the sensor was obtained by repeat SWV voltammetry and analysis of the voltammograms with a custom MATLAB script, affording a highly stable signal baseline with a temporal resolution of ≈4 s. Injection of anion aliquots (HSO_4_^−^, H_2_PO_4_^−^ or Cl^−^) into the flow then induced response spikes in the sensograms (Fig. 30B), the magnitudes of which were pleasingly identical to that obtained under standard, “static” conditions. Importantly, upon washing with fresh electrolyte, the sensor's response quickly returned to its baseline, confirming complete anion removal. Impressively, the sensor could be continuously operated over a 4.5 h period (corresponding to 3700 voltammetric scans), with a highly reproducible response to repeat HSO_4_^−^ injections and minimal baseline drift of ≤5 mV (Fig. 30C).

Surface-immobilisation of ion receptors is associated with a further unique advantage over solution-phase sensing; an ability to utilise other electroanalytical techniques. This most notably includes electrochemical impedance/capacitance spectroscopy (EIS/ECS). The former is well-established for the sensing of ions at redox-inactive receptive electrode surfaces, whereby a signal-generating solution-phase redox probe (typically ferri/ferrocyanide) is employed.^153–155^

Upon interfacial ion recognition, electrostatic interactions between the charged redox probe and the receptive surface are altered such that a change in the charge-transfer resistance is observed (Faradaic impedance).

In contrast, faradaic capacitance spectroscopy relies on changes in the capacitive, that is charge-storing, properties of a surface-bound redox transducer.^156^ This redox capacitance *C*_r_ is highly sensitive to changes in local (dielectric) properties and is well-established for biosensing.^157–159^

**Fig. 31:**
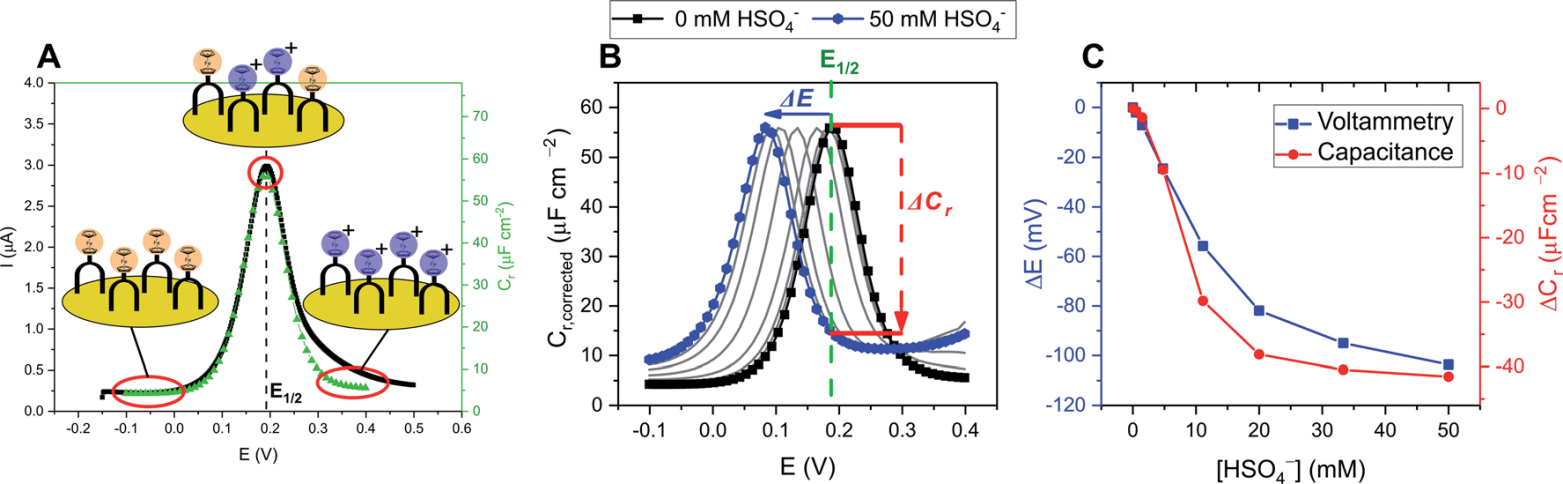
(A) Comparison of SWV (black) and redox capacitance *C*_r_ (green triangles) as a function of electrode potential (*i.e.* redox density of states (DOS)) of 28.XB_SAM_ in ACN/H_2_O 99 : 1. Schematically depicted is the ratio of oxidised and reduced anion receptors at different potential regimes whereby ferrocene/ferrocenium units are shown in orange and blue, respectively. (B) Normalised redox capacitance *C*_r_ as function of potential in response to increasing concentrations of HSO_4_^−^. The blue arrow indicates the “standard” potential shift as typically resolved voltammetrically. The dashed red arrow indicates the drop in *C*_r_ at *E*_1/2_ (dotted black line). (C) Comparison of voltammetric (blue) and redox capacitive response isotherms at *E*_1/2_ (red). Reproduced with permission from ref. 71 copyright 2021 American Chemical Society.

In 2021, Patrick and Hein *et al.* demonstrated for the first time the utility of this approach for ion sensing.^71^ This study was carried out on the same XB receptive interface 28.XB_SAM_, enabling a direct comparison with the afore-discussed voltammetric sensing format. Redox capacitance spectroscopy at a fixed AC frequency was employed to resolve the interfacial redox capacitance *C*_r_ which reports on the redox density of states (DOS) of the electro-active interface. Resolving this DOS (green triangles, Fig. 31A) affords, in the first instance, analogous information as standard SWV (black trace), *i.e.* it reports on anion binding-induced cathodic shifts (Fig. 31B).

However, in contrast to voltammetry, each point within this *C*_r_ DOS distribution is recorded at equilibrium, *i.e.* does not require a potential sweeping. Instead, *C*_r_ can be continually measured at a constant, freely chosen electrode potential and provides a simple, constant, direct sensor readout. For example, if *C*_r_ is continually monitored at the initial *E*_1/2_ of the interface then anion binding induces a drop in signal, as *C*_r_ now lies in the anodic tail of the redox distribution. The sensing isotherm obtained in this manner is similar to that obtained by standard voltammetry (Fig. 31C), however the binding and response are somewhat enhanced due to a self-amplification effect in the redox capacitive format.

**Fig. 32:**
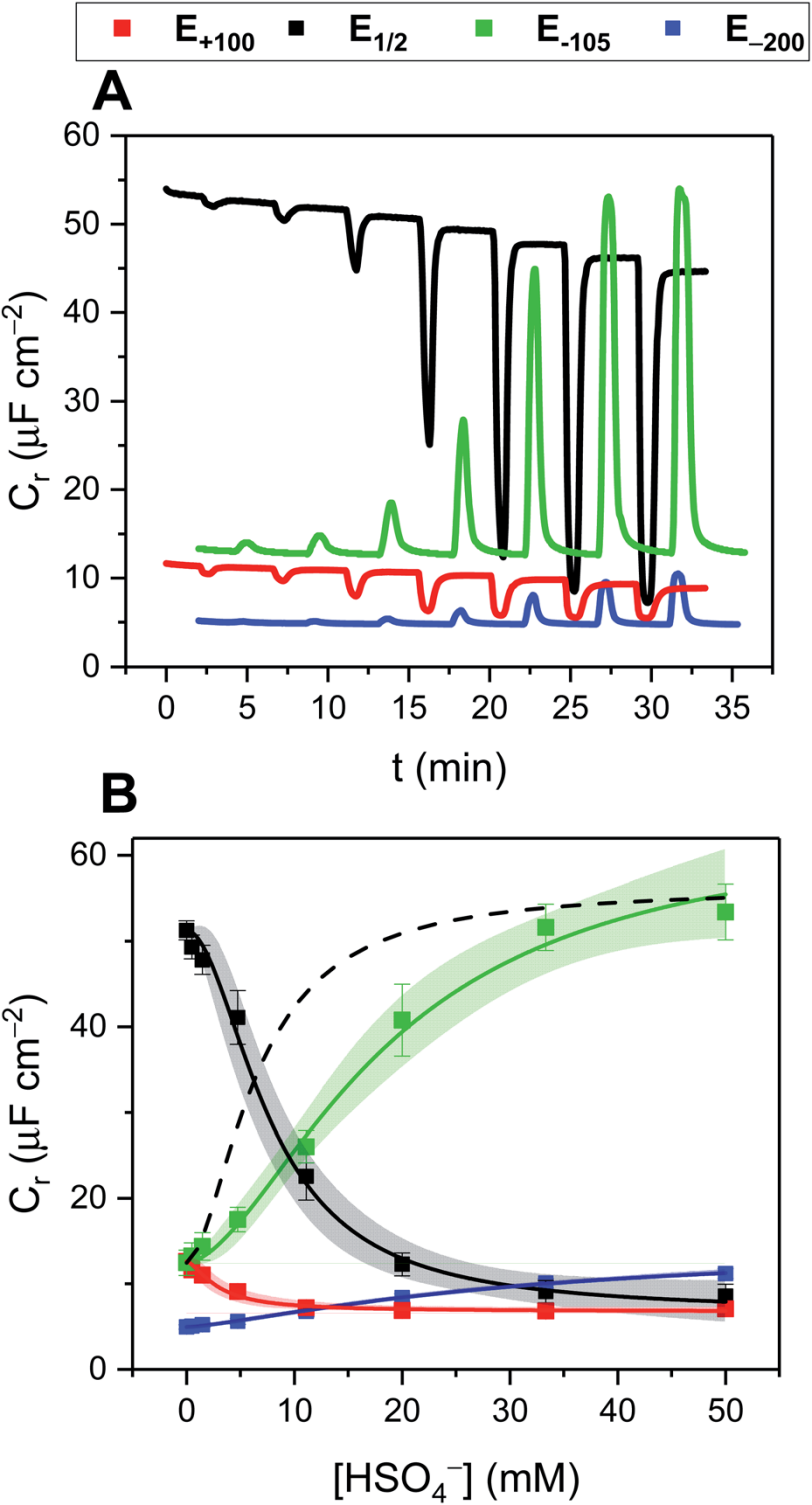
(A) Redox capacitance response of 28.XB_SAM_ towards HSO_4_^−^ at *E*_1/2_ (black), *E*_−105_ (green), *E*_−200_ (blue) and *E*_+100_ (red) under continuous electrolyte flow in a custom 3D-printed flow cell (see Fig. 31A). Each spike in (A) represents the response towards aliquots of HSO_4_^−^ of increasing concentrations with absolute signal increasing or decreasing depending on the initial surface polarisation. (B) The corresponding baseline-corrected response isotherms. The dashed black line represents, for simpler comparison, the mirrored capacitance response at *E*_1/2_, highlighting the steeper response slope and enhanced anion binding magnitude at *E*_1/2_*vs.* at *E*_−105_. Reproduced with permission from ref. 71 copyright 2021 American Chemical Society.

As a result of its direct readout, necessitating no further data analysis, and its high temporal resolution (≈2.5 s) this novel methodology is ideally suited for real-time, continuous flow ion sensing, as investigated in the same 3D-printed flow cell as used for previous voltammetric studies. As shown in Fig. 32A, injection of HSO_4_^−^ aliquots of increasing concentration induced noticeable response spikes, whose magnitude and “direction” are dependent on the applied electrode potential. In addition to this signal switch-on/off control the apparent binding constant (*i.e.* “steepness”) of the response isotherm can be modulated by judicious choice of potential (Fig. 32B) by up to 1 order of magnitude (*K*_app_ at +100 mV = 320 M^−1^*vs.* 36 M^−1^ at −200 mV). Concomitantly, the sensor's LOD can be tuned in this manner, and is with ≈45 μM (for measurements at *E*_1/2_), lower than in an optimised voltammetric format,^72^ which cannot be additionally tuned. With a higher temporal resolution, direct sensor readout as well as improved and tuneable analytical performance, this redox capacitive sensing format is thus vastly superior to standard voltammetry. In addition to supporting improved ion sensing capabilities, preliminary investigations suggest that the redox capacitive readout maybe used to elucidate interfacial host–guest binding kinetics and thus also presents a novel tool in the fundamental study of interfacial electro-active supramolecular host–guest systems.

### 4.2 Other electrochemical sensors

#### 4.2.1 Capacitive sensors

Electrochemical capacitance/impedance spectroscopy can also be carried out in an entirely non-faradaic format, that is in the absence of either a solution-phase or surface-bound redox probe. In this case the interfacial non-faradaic capacitance of receptor-modified electrodes can serve as a transducer for an ion binding event, which is well-established for the sensing of cations at crown-ether modified electrodes.^160,161^

**Fig. 33:**
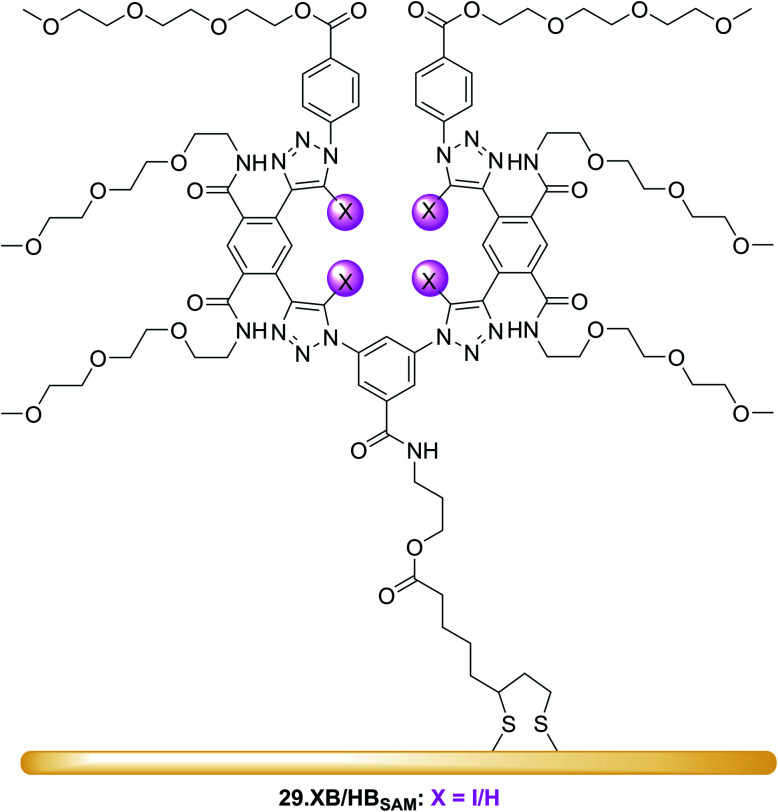
XB and HB foldamer SAMs for anion sensing in pure water *via* non-faradaic capacitance spectroscopy.

Exploiting the uniquely potent performance of XB for anion sensing in water, Hein *et al.* recently demonstrated, for the first time non-faradaic capacitive anion sensing at XB and HB foldamer molecular films 29.XB/HB_SAM_ (Fig. 33).^162^

**Fig. 34:**
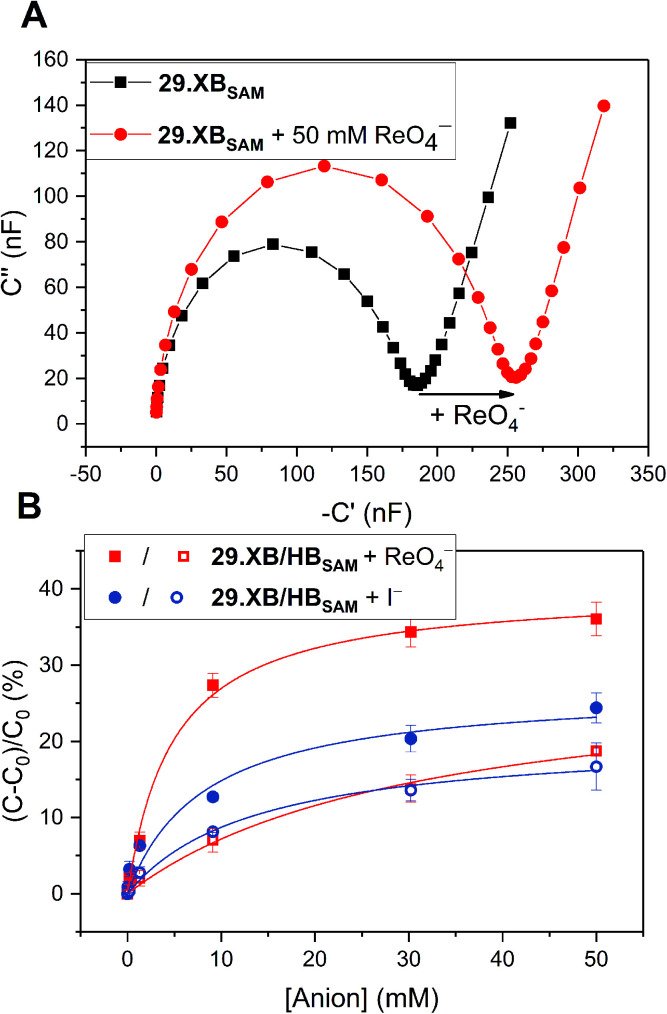
(A) Capacitive Nyquist plots of 29.XB_SAM_ in the absence and presence of 50 mM ReO_4_^−^ showing an increased capacitance in the presence of bound anion. (B) Relative capacitive sensor response of 29.XB_SAM_ (filled symbols) and 29.HB_SAM_ (empty symbols) in response to ReO_4_^−^ (red) and I^−^ (blue). Reproduced from ref. 162 with permission of the Royal Society of Chemistry.

In pure water this sensor responded selectively to the environmentally and biologically relevant charge-diffuse anions ReO_4_^−^, I^−^ and SCN^−^ by an increase in the interfacial capacitance (Fig. 34A) while neither Cl^−^, Br^−^ nor ClO_4_^−^ induced any response. This is notably different to the recognition behaviour of the parent foldamer receptor in solution-phase, where 2 : 1 host–guest stoichiometric binding with ReO_4_^−^, I^−^, SCN^−^, Br^−^ and ClO_4_^−^ was ascertained *via* isothermal titration calorimetry (ITC), with *β* = *K*_1_*K*_2_ of up to 1.45 × 10^10^ M^−2^ for I^−^. In contrast, binding was significantly attenuated at the surface for 29.XB/HB_SAM_ (*K* ≤ 360 M^−1^), and proceeds *via* formation of 1 : 1 host–guest complexes, as suggested by interfacial binding isotherm analysis according to the Langmuir adsorption model. As representatively shown in Fig. 34B, the XB sensor 29.XB_SAM_ outperformed its HB congener in all cases, with not only higher maximum signal magnitudes but also increased binding strength.

The binding of ReO_4_^−^ in particular was enhanced significantly for 29.XB_SAM_ (*K* = 231 M^−1^) in comparison to 29.HB_SAM_ (*K* = 11 M^−1^).

Relatedly, the LOD of 29.XB_SAM_ was ≈3-fold improved in all cases and was lowest for I^−^ (14 μM). The physicochemical origins of these capacitive sensor response patterns were also later analysed and justified within a mesoscopic model.^163^ Of note is that, in principle, this sensor can, akin to the redox capacitive sensor 28.XB_SAM_, be operated at a fixed frequency and freely chosen electrode potential and may thus be used for real-time flow anion sensing in pure water.

#### 4.2.2 Potentiometric sensors

The most generically applicable commercial real-life relevant ion sensing methodology relies on the potentiometric determination of ions using ion-selective electrodes (ISEs).^49,164,165^ They respond to ingress of the analyte ion into an ion-selective membrane *via* a change in the electromotive force (*i.e.* a potential change) between the membrane-containing ISE and a reference electrode. In an ideal case the response of the ISE is, according to Nernstian principles, determined by *E* = *E*_0_ + *S* log *α*_i_, where *E*_0_ is a constant, *α*_i_ the activity of the ion and 
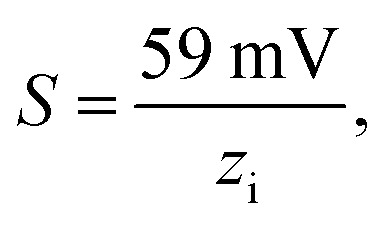
 where *z*_i_ is the charge of the ion. For a monovalent ion an ideal “Nernstian” response of 59 mV per decade change of ion concentration is thus expected. In order to render the ISE selective toward the target analyte, ion receptors (ionophores) are typically incorporated into the hydrophobic membrane component of the ISE. To date, the vast majority of anion ISEs rely on Lewis acid metal complexes or traditional HB receptors as ionophores.^40^ Surprisingly, σ–hole receptors have, despite their typically contrasting selectivity patterns and larger hydrophobicity, only very recently been explored in ISEs. Specifically, the first, and thus far only, example of a XB ionophore 30.XB was reported by Lim, Goh and co-workers for the potentiometric sensing of iodide in pure water.^166^

Initial ^1^H NMR studies in d_6_-acetone indicated convergent XB mediated I^−^ recognition with moderate 1 : 1 host–guest stoichiometric binding of *K* = 260 M^−1^. In contrast, I^−^ binding of the HB analogue 30.HB was significantly attenuated with *K* = 2.75 M^−1^. Incorporation of the ionophores into polymeric membranes of varying composition produced a series of ISEs, all of which responded with a near-Nernstian response of ≈50 mV/decade and a LOD of ≈1.25 μM to iodide. The authors then conducted a range of selectivity studies in the presence of the potentially interfering anions Cl^−^, Br^−^, NO_3_^−^, SCN^−^ and ClO_4_^−^. Based on the Hofmeister series, anions of low hydrophilicity, *i.e.* more hydrophobic ones, can more easily ingress into the membrane to induce the largest interference, as observed for a control membrane without ionophore, which displayed the expected selectivity pattern of: ClO_4_^−^ > SCN^−^ > I^−^ > NO_3_^−^ > Br^−^ > Cl^−^.

While incorporation of the 30.HB ionophore into the membrane did not appreciably alter this selectivity trend, the XB ionophore induced moderate enhancements in I^−^ selectivity over all other tested anions with a notable, modest, preference for I^−^ over SCN^−^, indicating that specific XB mediated I^−^ recognition takes place within the membrane.

**Fig. 35:**
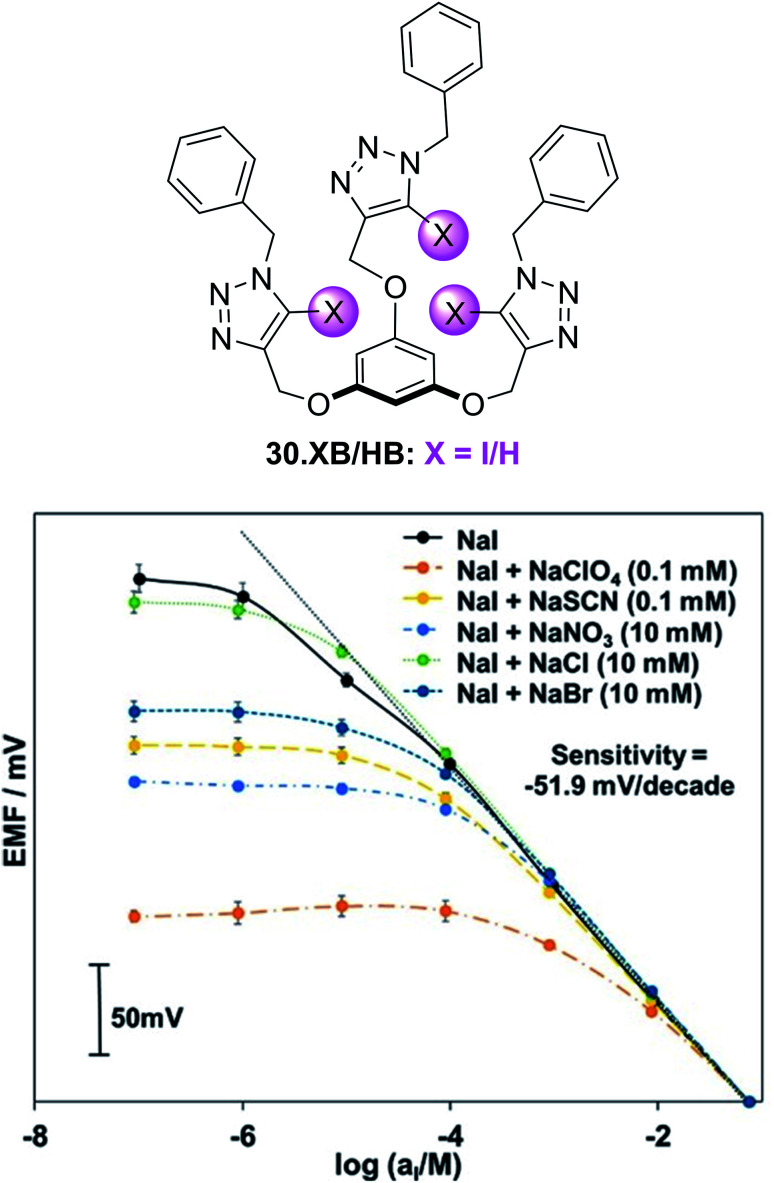
Response of an ISE containing ionophore 30.XB toward iodide in the absence of presence of competing anions. Reproduced with permission from ref. 166 copyright 2021 American Chemical Society.

As shown in Fig. 35, the I^−^ selectivity over the more hydrophilic halides was sufficiently large such that 10 mM Cl^−^ or Br^−^ did not interfere. In contrast, the more hydrophobic SCN^−^ and in particular ClO_4_^−^ significantly interfered with I^−^ determination at much lower levels of 0.1 mM. Nevertheless, this study provides an important first foray into the exploitation of σ–hole interactions in ISEs which will undoubtedly receive more attention in the future, in particular for the sensing of softer anions.

## 5. Other sensors

Akin to electrochemical sensors, chemiresistive sensors have emerged as simple, cheap and scalable sensing devices.¶¶While very similar, chemiresistors are technically not electrochemical devices, as no chemical change is occurring as a result of current/voltage perturbations. However, this differentiation is generally not relevant and chemiresistors usually possess the advantages that are associated with typical electrochemical techniques. They respond to analyte presence by changes in the conductance of a sensing material immobilised between two electrodes, a concept that has been exploited in particular for sensing of gases,^167^ but also ions.^168–170^

**Fig. 36:**
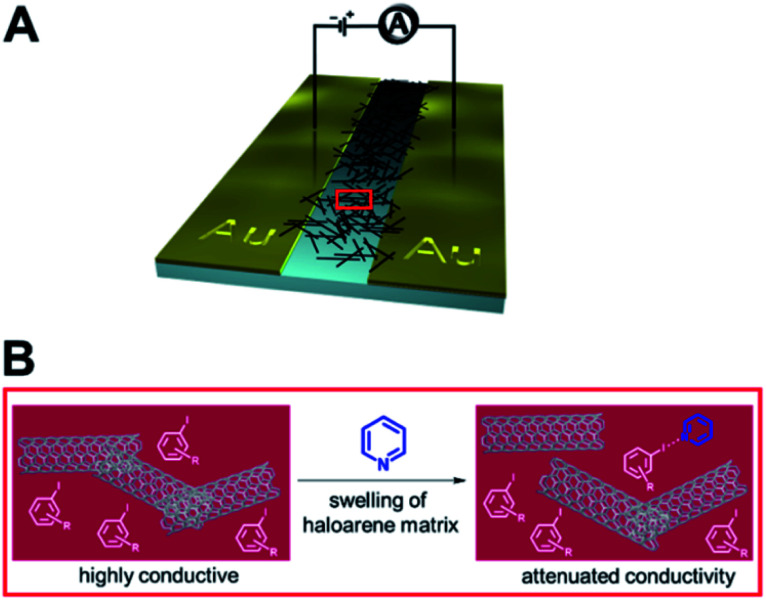
(A) Schematic depiction of a chemiresistive sensor based on a carbon nanotube matrix between two gold electrodes. (B) Incorporation of haloaryl XB selectors into the sensor matrix enables sensing of pyridine vapours by recognition-induced swelling of the matrix and an associated decrease in conductance. Reproduced with permission from ref. 171 copyright 2016 American Chemical Society.

In 2016, the group of Swager demonstrated for the first time the utility of XB “selectors” as host motifs to enable selective chemiresistive sensing of pyridine gas.^171^ To this end they modified single-walled carbon nanotubes (SWCNTs) with haloaryl XB hosts by solvent-free ball-milling. The resulting selector-modified SWCNTs were then immobilized between gold electrodes and their conductance G measured in the absence and presence of pyridine (Fig. 36A).

**Fig. 37:**
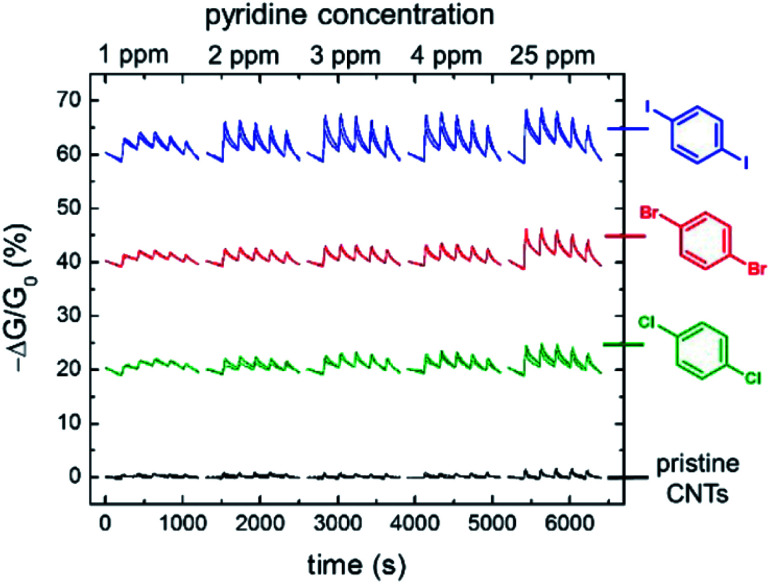
Conductance response of chemiresistive gas sensors containing different dihaloaryl XB selectors in the repeating presence of increasing concentrations of pyridine in N_2_ carrier gas. For clarity the relative conductance responses are offset by 20%. Reproduced with permission from ref. 171 copyright 2016 American Chemical Society.

As shown in Fig. 37, *p*-dihalobenzene selectors enabled sensing of low concentrations of pyridine gas (<25 ppm) by a decrease in electrical conductance, induced by swelling of the sensing matrix (Fig. 36B). Importantly, the *p*-diiodobenzene host enabled more sensitive sensing than the bromo or chloro-congeners, consistent with a sensor response arising from XB-mediated recognition.

Specifically, the *p*-diiodobenzene-containing sensor displayed the largest response of −Δ*G*/*G*_0_ = 5.1 ± 0.9% in response to only 3 ppm pyridine, while much higher pyridine concentrations were required to induce significant responses when the other selectors were employed. Further evidence for the crucial role of XB in this sensor was also obtained by experiments using iodo- or bromodurene as mono-haloaryl selectors as well as studies with 4-methylpyridine as analyte, which due to its enhanced Lewis basicity induced larger responses. Of further note is the response of these XB sensors displaying a relatively high level of selectivity; depending on the selector, even very high concentrations (>1000 ppm) of the potential interferents acetonitrile, benzene, isopropanol or hexanes induced only minor changes in conductance.

Similar chemiresistive gas sensors based on various *p*-dihalobenzene XB selectors and a SWCNT matrix were also developed for the detection of cyclohexanone and dimethyl-dinitro-butane (DMNB).^172^ These analytes were chosen as model compounds for detection of nitro-containing explosives, the latter also serving as a tagant or marker compound in certain plastic explosives. Unsurprisingly, the diiodoaryl based selectors outperformed their bromo-counterparts for the sensing of cyclohexanone, with linear conductance decreases of up to 12%. Initial experiments indicate that the sensor can also detect the less volatile DMNB. A variety of other analytes also induced significant responses (EtOH, ACN, EtOAc, cyclohexane and acetone), however this was attributed to their much higher vapour pressures and thus higher concentrations under the experimental conditions.

Notably in both of these studies, the sensor was easily regenerated by exposure to pure N_2_ gas, confirming the reversibility of the analyte–sensor interaction and enabling facile sensor re-use.^171,172^

**Fig. 38:**
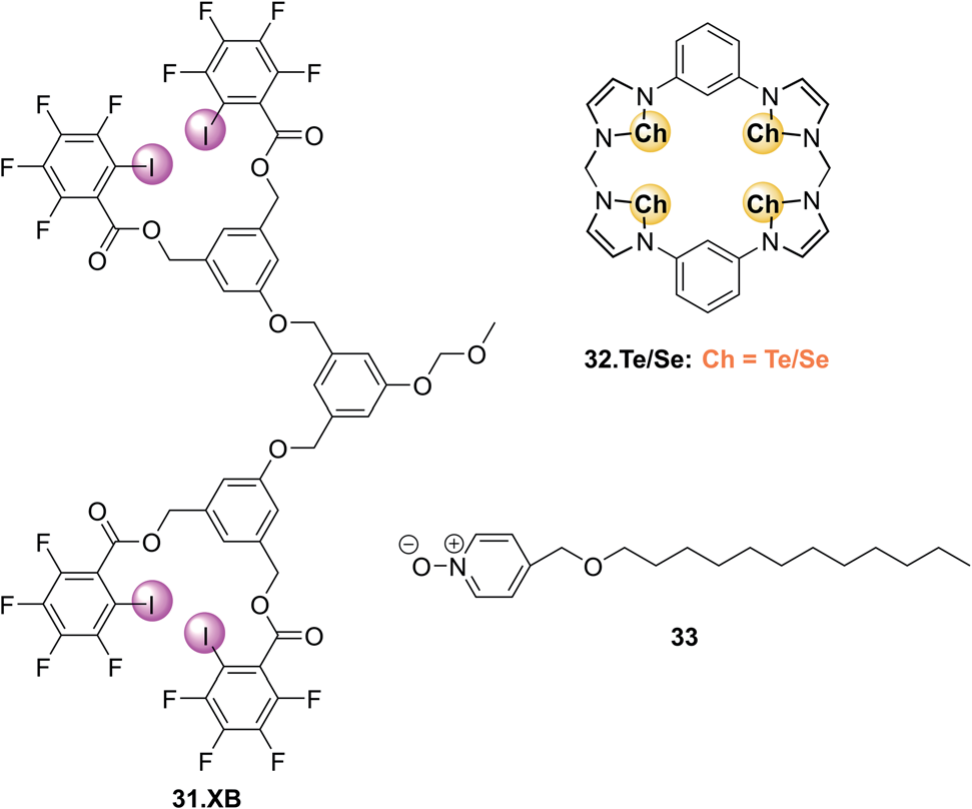
Chemical structures of XB organogelator 31.XB, a visual Cl^−^ sensor by anion induced gel–sol transition and ChB quasi-calix[4]-chalcogenadiazole hosts 32.Te/Se which upon interaction with surfactant 33 form supramolecular fibres or vesicles in water, respectively. Cl^−^ or Br^−^ induced disassembly of 32.Te-33 vesicles releases the fluorescent doxorubicin cargo, presenting a stimuli-responsive drug carrier and indirect optical halide sensor.

In 2016 Liu *et al.* reported a XB organogelator 31.XB containing multiple peripheral iodoperfluoroarene moieties as a visual and rheological Cl^−^ sensor (Fig. 38).^173^

In acetone/hexane 1 : 8 the free gelator formed an organogel, which collapsed into a solution within 10 min upon addition of 0.8 equiv. Cl^−^. In contrast, the same amount of Br^−^ only induced a small degree of gel–sol transition while HSO_4_^−^, NO_3_^−^, CN^−^ or I^−^ had no effect on the gel. Only at higher concentrations (2 equiv. for Br^−^ and 5 equiv. for I^−^) was dissolution of the gel achieved. These observations are in good agreement with the anion association constants of the gelator, determined by ^1^H NMR titrations in acetone, which were largest for Cl^−^ (650 M^−1^) and smaller for Br^−^ (390 M^−1^) and I^−^ (140 M^−1^), while the other anions did not bind significantly.

A similar anion binding-induced transformation of a supramolecular assembly was also reported based on ChB quasi-calix[4]-chalcogenadiazole hosts 32.Te and 32.Se, which upon interaction with a pyridine *N*-oxide surfactant 33 in water self-assembled into vesicle or nanofibers, respectively (Fig. 38).^174^ Upon exposure to halide anions, or lowering the pH, these formations disassembled. In the case of the vesicles formed by 32.Te and 33, this was exploited as a proof-of-principle for release of the chemotherapeutic doxorubicin (DOX). Specifically, DOX-loaded vesicles were ruptured by addition of Cl^−^ or Br^−^, resulting in DOX release and increase of DOX fluorescence, thereby presenting an indirect halide sensor.

## 6. Conclusions and outlook

The considered employment of sigma–hole interactions in the development of sensors, in particular for anions, but also neutral (gaseous) Lewis basic analytes, has significantly matured in the last decade. This includes a large range of both optical and electrochemical sensing approaches, in particular those based on fluorescent as well as voltammetric readouts. In addition, various other sensor formats, most notably chemiresistors as well as (redox)capacitive sigma–hole sensors have been developed recently. In all these formats an improved performance of the sigma–hole sensor (in particular XB) in comparison to a structurally analogous HB sensor is typically observed, including enhanced response magnitudes and sensitivities, lower LODs and/or enhanced/altered selectivity patterns. This arises as a result of enhanced binding magnitudes and/or enhanced signal transduction, which in turn can be attributed to the inherent characteristics of sigma–hole interactions, most notably lower solvent dependencies, higher hydrophobicities, stricter geometric binding preferences and altered thermodynamic binding contributions. As a result of these combined advantages, increasingly sophisticated and potent XB sensors have been developed, in particular in the last ≈5 years. This includes, for example, systems capable of anion sensing in increasingly competitive media, including pure water,^101,162,166^ as well as continuous, real-time sensing systems.^71,72,131,171,172^

These significant advances in a comparably short amount of time attest to the enormous future potential of sigma–hole based sensors for real-life relevant applications and provide an excellent foundation for a broad range of research activities in sensor development and related applications as well as fundamental host–guest studies. We believe further efforts in this field will/should focus on the following:

### 6.1 Sensing in aqueous media

In spite of the aforementioned examples, anion sensing in predominantly aqueous media remains a highly important but formidable challenge, which can, in part, be attributed to the significant synthetic complexity of receptive, water-soluble probes. This can partially be circumvented by use of non-diffusive sensing formats (*e.g.* surface immobilisation) thereby negating the need for solubilising groups. Similarly, the omission of reporter groups can reduce synthetic complexity, but this can only be done if a sensing approach without a transducer is used, *e.g.* in potentiometric, chemiresistive or impedimetric/capacitive formats.

### 6.2 Fundamental studies into and exploitation of (other) sigma–hole interactions

While XB and, to a lesser extent ChB based sensors, are established, the application of pnictogen bonding (PnB) and tetrel bonding (TrB) as non-covalent supramolecular interactions for sensing has not been reported to date. Nevertheless, there is an increasing interest in the design and application of such receptors,^14,16,175–177^ and it is expected that they would display potent sensory performance. In light of the recently developed electroactive ChB systems^62^ it appears that redox-control of PnB or TrB may be a particularly promising approach to not only generate novel sensors but to also gain fundamental insights into their intrinsic bonding properties.^177^ Such fundamental investigations have already been carried out on a range of XB systems, in particular *via* voltammetric methodologies,^54,125–128^ which allow for the transient reversible generation of a differently charged (potentially not otherwise accessible) species. This enables simultaneous investigation of the sigma–hole properties of a receptor in multiple redox states, an approach that will undoubtedly prove useful in a continued investigation of sigma–hole properties.

### 6.3 Development of novel sensing approaches and mechanisms

We envision a further exploration of recently developed or (in the context of XB/ChB-mediated recognition) underexplored methodologies such as impedimetric or (redox)capacitive methodologies,^71,162^ chemiresistive^171,172^ or potentiometric approaches.^166^ In addition, other sensing principles are ripe for exploration in concert with sigma–hole interactions, such as indicator displacement assays (IDAs)^178^ or the recently developed transporter–liposome–fluorophore (TLF) approach which relies on the quenching of a vesicle-encapsulated fluorophore by the analyte ion.^179^ In the latter, a selectivity enhancement is achieved by use of a transmembrane ion transporter, such that only ions which can cross the vesicle membrane *and* quench the encapsulated fluorophore induce a response. Due to their typically improved or contrasting anion transport selectivities and efficacies, sigma–hole anionophores have much to offer as potent anionophores in TLF assays.^33–36,175,180^

### 6.4 Device and materials integration

As highlighted in Section 2.3, sensor integration into condensed matter, in particular surfaces, membranes and polymeric architectures will be an indispensable avenue towards enabling many real-life relevant sensing applications such as flow sensors and microfluidic devices, as only in these formats the main advantage of the non-covalent sensing approach, its reversibility, can be exploited.^68,71,72,181^ In addition to sensor re-usability and more facile device integration, this is associated with various other benefits, including surface enhancement effects, circumventing solubility constraints, and the use of otherwise inaccessible sensing formats/readouts (*e.g.* impedance/capacitance/chemiresistance).^40,137,151^

In spite of the enormous potential, surface immobilisation or material-integration sigma–hole mediated sensing remains comparably underdeveloped, and is only established in electrochemical^57,71,72,137,152,162^ and chemiresistive formats.^171,172^ Interfacial XB or ChB optical sensors remain even more embryonic; the only example being the benzoselenadiazole fibres developed by Che for fluorescent gas sensing.^131^ Clearly there is significant untapped potential in further exploration of XB and ChB (sensing) materials,^182,183^ particularly in thin films and (interfacial) polymers^184–186^ in electrochemical, optical and other sensing formats.

## Author contributions

RH and PDB co-wrote/edited the review article.

## Conflicts of interest

There are no conflicts to declare.

## Supplementary Material
